# Direct Visualization of a Hydroperoxy Intermediate in Fluorescent Protein Chromophore Maturation in PAmCherry1

**DOI:** 10.1021/jacs.6c13605

**Published:** 2026-09-21

**Authors:** Hanifa Bibi, Leonie De Vrieze, Elke De Zitter, Nicolas Coquelle, Janko Civic, Eduard Fron, Kenichi Kamata, Masaya Usui, Kaori Otsuki, Gilles Garré, Ronald Rios-Santacruz, Giorgio Schirò, Fangjia Luo, Nipawan Nuemket, Jungmin Kang, Shigeki Owada, Kensuke Tono, Thi Yen Hang Bui, Olivia Olislaegers, Hidetsugu Asada, Dohyun Im, Luc Van Meervelt, So Iwata, Nobuhiko Miyasaka, Jeremy N. Harvey, Eriko Nango, Martin Weik, Hideaki Mizuno

**Affiliations:** † Laboratory of Biomolecular Network Dynamics, Biochemistry, Molecular and Structural Biology Section, Department of Chemistry, 26657 KU Leuven, Celestijnenlaan 200G bus 2403, 3001 Leuven, Belgium; ‡ 55543 Université Grenoble Alpes, CEA, CNRS, Institut de Biologie Structurale, 38000 Grenoble, France; § Quantum Chemistry and Physical Chemistry Section, Department of Chemistry, KU Leuven, Celestijnenlaan 200F bus 2404, 3001 Leuven, Belgium; ∥ Molecular Imaging and Photonics, Department of Chemistry, KU Leuven, Celestijnenlaan 200F bus 2404, 3001 Leuven, Belgium; ⊥ College of Science and Technology, 12976 Nihon University, 1-5-1 Kandasurugadai, Chiyoda-ku, Tokyo 101-0062, Japan; # Support Unit for Bio-Material Analysis, RIKEN Center for Brain Science, 2-1 Hirosawa, Wako-city, Saitama 351-0198, Japan; g 133704 Japan Synchrotron Radiation Research Institute, 1-1-1 Kouto, Sayo, Hyogo 679-5198, Japan; h 73559 RIKEN SPring-8 Center, 1-1-1 Kouto, Sayo-cho, Sayo-gun, Hyogo 679-5148, Japan; i Faculty of Chemistry, Hanoi National University of Education, 136 Xuan Thuy street, Cau giay district, Hanoi 123105, Vietnam; j Department of Cell Biology, Graduate School of Medicine, 38049 Kyoto University, Yoshidakonoe-cho, Sakyo-ku, Kyoto 606-8501, Japan; k Laboratory of Biomolecular Architecture, Biochemistry, Molecular and Structural Biology Section, Department of Chemistry, KU Leuven, Celestijnenlaan 200G bus 2403, 3001 Leuven, Belgium; l Institute of Multidisciplinary Research for Advanced Materials, 13101 Tohoku University, Aoba-ku, Sendai 980-8577, Japan

## Abstract

Despite the widespread use of fluorescent proteins in bioimaging, the mechanism of oxidative chromophore maturation to form the tyrosine C_α_=C_β_ double bond remains unclear. In the photoactivatable fluorescent protein PAmCherry1, this double bond has not yet been formed prior to photoactivation. Here, serial femtosecond crystallography (SFX) using an X-ray-free electron laser (XFEL) enabled us to capture the radiation damage-free structure of non-photoactivated PAmCherry1. This structure displayed a covalently bound hydroperoxy moiety (73% occupancy) at the sp^3^-hybridized C_α_ atom of the chromophore tyrosine residue within the imidazolinone ring. A unique hydrogen-bonding network surrounding the hydroperoxy moiety stabilizes this highly reactive chromophore species. Electrospray ionization mass spectrometry (ESI-MS) supported the coexistence of hydroperoxy and enolate chromophore forms. According to spectroscopic analysis, these species exist in dynamic equilibrium. MS analysis further demonstrated that photoactivation is accompanied by the formation of the mature chromophore containing a C_α_=C_β_ double bond together with Kolbe decarboxylation. Time-resolved SFX (TR-SFX) using a femtosecond pump laser further revealed decreasing electron density at 800 ps after photoexcitation, consistent with rapid oxidation and decarboxylation. Collectively, these results support a hydroperoxy intermediate as a key species in oxidative chromophore maturation, which is stabilized in non-photoactivated PAmCherry1.

## Graphical abstract

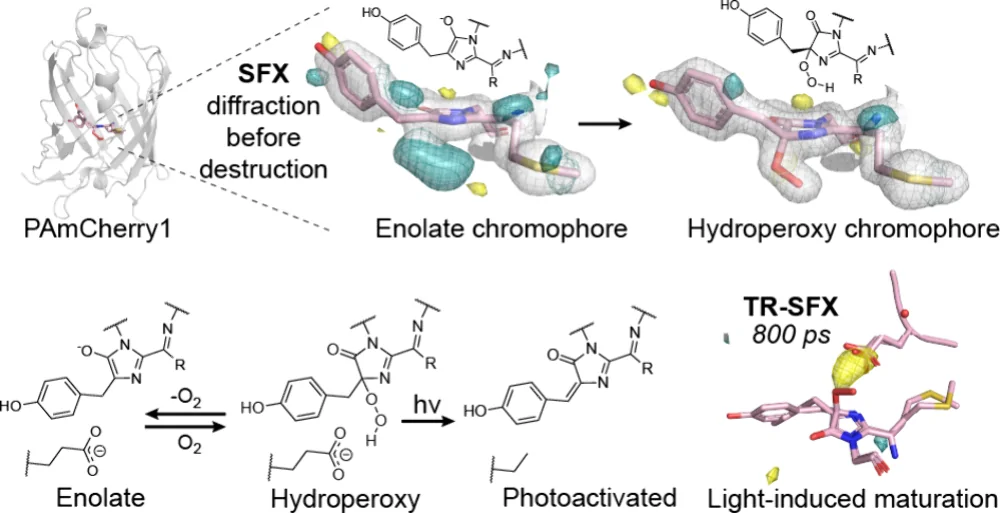

Fluorescent proteins have revolutionized molecular labeling in living cells through autocatalytic chromophore formation. This process proceeds via cyclization initiated by nucleophilic attack, followed by dehydration and oxidation (Scheme 1).^1^ The oxidation step requires molecular oxygen and generates hydrogen peroxide stoichiometrically, yet its precise mechanism remains elusive.^2,3^ A proposed mechanism involves electron transfer from the enolate form of the imidazolidinone at Cα66 (corresponding to Cα67 in PAmCherry1)^2,4–6^ or C’65 ^7^ to molecular oxygen, leading to the formation of a hydroperoxy adduct. Notably, this mechanism is inferred from observations of mutants that fail to mature into a functional fluorescent protein^2,4–6^ or a FP variant of which the chromophore is derived from p-azido-l-phenylalanine, a noncanonical amino acid.^7^ To date, no hydroperoxy adduct has been directly observed.

**Scheme 1:**
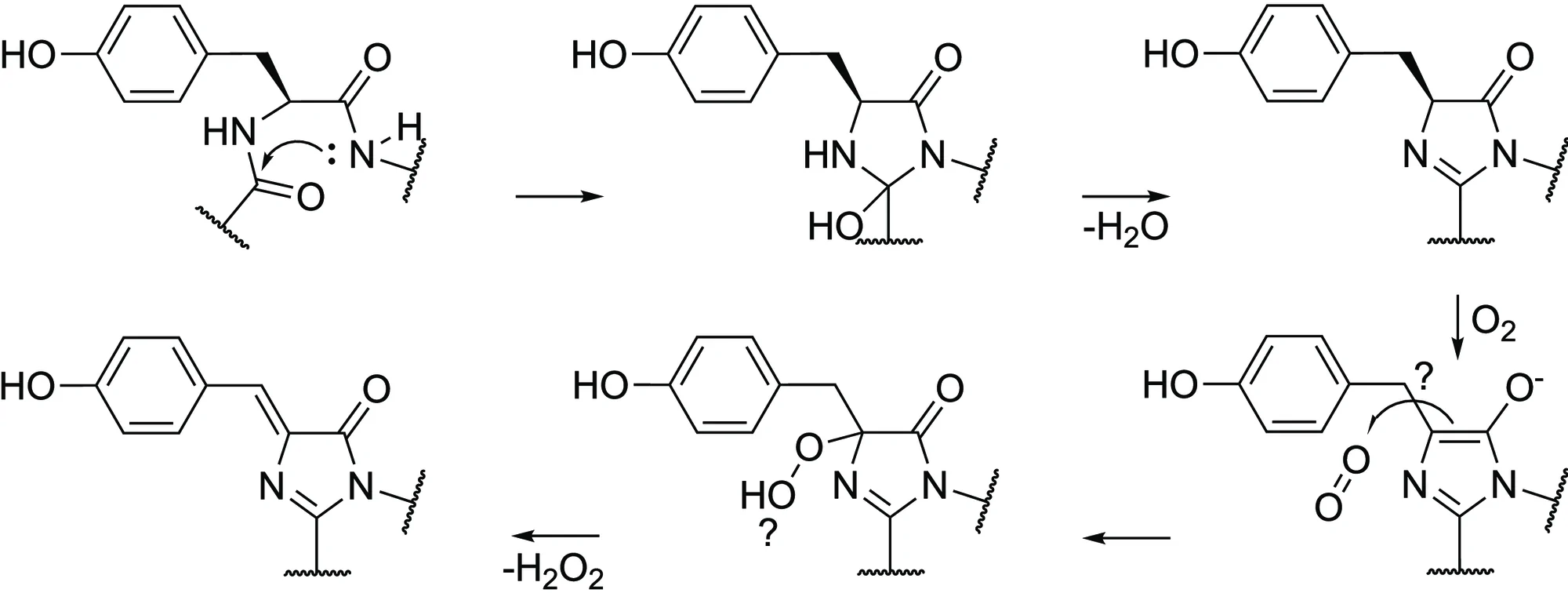
Proposed Chromophore Formation Process of Fluorescent Proteins

To address the oxidation mechanism, we employed the photoactivatable fluorescent protein PAmCherry1 as a model.^8^ According to the reported crystal structure, the oxidation of the chromophore at Cα67-Cβ67 is not yet completed in the non-photoactivated state (PDB: 3KCS), and a Cα67=Cβ67 double bond is formed concomitantly with the Kolbe-type decarboxylation of E215 upon photoactivation (PDB: 3KCT).^9^ In this study, we determined the crystal structure using serial femtosecond crystallography (SFX) with an X-ray free electron laser (XFEL), thereby avoiding radiation damage through “diffraction before destruction”.^10^ The SFX-based structure revealed unprecedented electron density corresponding to a Cα67-hydroperoxy imidazolinone, which has not been observed in synchrotron-based structures,^9^ most likely due to X-ray radiation damage. These findings provide direct structural evidence for a previously uncharacterized oxidation intermediate in PAmCherry1 and allow a more detailed investigation of the mechanism for chromophore oxidation in fluorescent proteins.

We first determined the crystal structure of PAmCherry1 in its non-photoactivated state using synchrotron radiation to provide a reliable reference for subsequent SFX experiments (Figure S2).^11^ The overall structure exhibited a typical 11-stranded β-barrel fold surrounding the chromophore, 4-acylimine-2-(4-hydroxybenzyl)­imidazolinone. The chromophore was observed in the enolate form, in which the anionic oxygen (O67) is stabilized by the guanidinium nitrogen of the R95 side chain through a 2.8 Å hydrogen bond. Furthermore, reduced electron density was observed at the E215 carboxylate, the site of photoactivation-associated Kolbe decarboxylation, suggesting partial X-ray-induced radiation damage (Figure S2).

To obtain a radiation damage-free structure, we subsequently performed SFX using an XFEL (Figure 1).^12,13^ Initial refinement of the SFX data using the synchrotron-derived enolate model revealed a prominent positive m*F*_obs_-D*F*_cal_ electron density peak adjacent to Cα67, at a position consistent with tetrahedral sp^3^ geometry (Figure 1b). To test whether this density corresponds to a hydroperoxy adduct at Cα67, we refined a model containing the hydroperoxy moiety and evaluated its agreement with the experimental data based on the residual electron density. The positive signal disappeared completely and only a faint negative electron density peak appeared around the proximal oxygen (O_proximal_) of the hydroperoxy moiety (Figure 1c). This negative electron density peak was accounted for by refinement using a two-state model comprising the enolate- and hydroperoxy-form chromophores, yielding occupancies of 27% and 73%, respectively (Figure 1d). We concluded that SFX revealed a hydroperoxy form of the non-photoactivated PAmCherry1 chromophore that coexists with an enolate form while the hydroperoxy form could not be captured by synchrotron-based crystallography.

**Figure 1:**
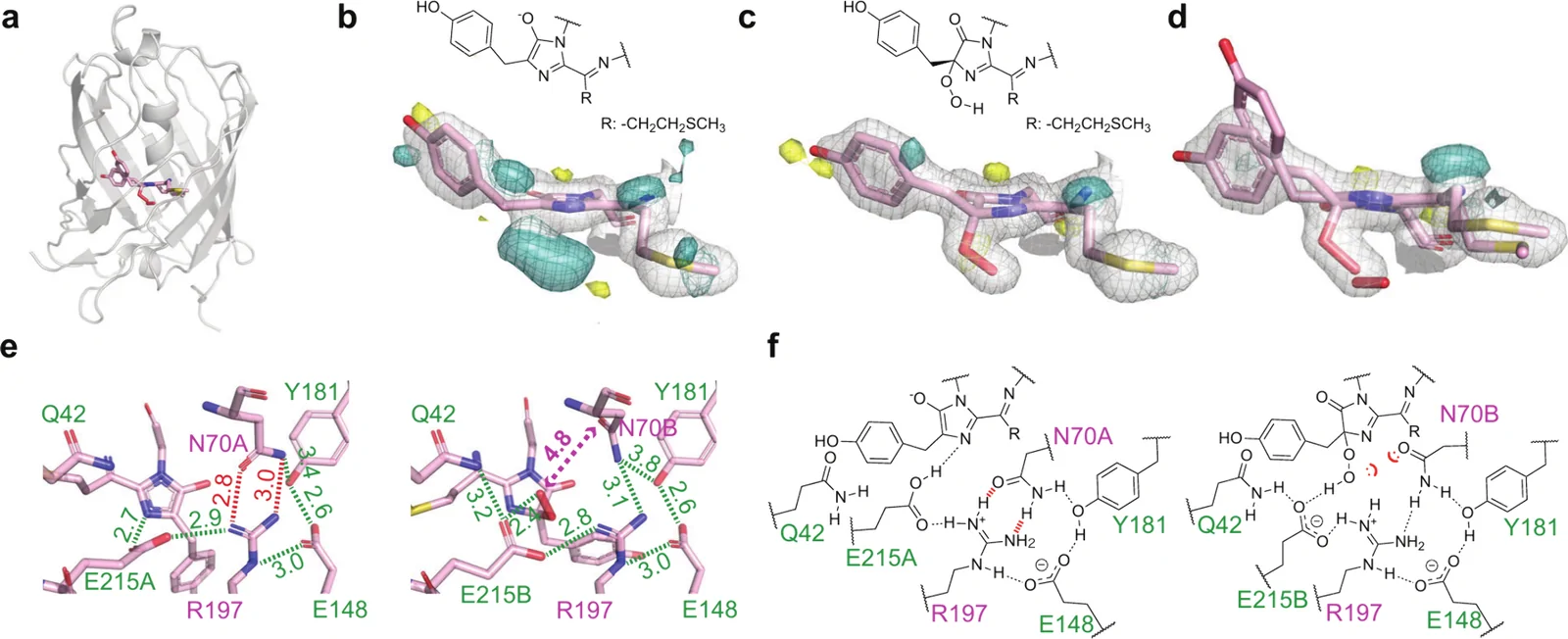
Hydroperoxy form chromophore of PAmCherry1 detected by SFX. (a) Overall structure of PAmCherry1 determined by SFX shown in a cartoon representation, with the chromophore highlighted as light pink sticks. Only one monomer (monomer A) is shown for clarity; the structure of the other monomer (monomer B) is provided in Figure S3. (b–d) Chromophore structures refined using an enolate form model (b), a hydroperoxy form model (c), or a two-state model comprising an enolate form chromophore with a modeled molecular oxygen and a hydroperoxy form chromophore (d). In the two-state model, the occupancies refined to 27% for the enolate-form and 73% for the hydroperoxy-form. The 2m*F*_obs_-D*F*_cal_ (gray, 1.0σ) and m*F*_obs_-D*F*_cal_ (yellow = negative/teal = positive, ±3.0σ) maps around the chromophore are shown. Chemical structures of the enolate and hydroperoxy form chromophores are shown above. (e) Hydrogen-bonding interactions surrounding the chromophore in the enolate (left) and hydroperoxy form (right). For clarity, the oxygen molecule refined adjacent to the chromophore in the enolate form is not shown. Amino acid residues unique to PAmCherry1 (N70 and R197) are labeled in magenta. Other surrounding residues (Q42, E148, Y181, E215) are indicated in green. N70 and E215 adopt two conformations, labeled A and B for the enolate and hydroperoxy forms, respectively. Distances between nearby oxygen and nitrogen atoms are shown with dotted lines, and the corresponding distances are indicated in Å. Green lines indicate hydrogen-bonding interactions, red lines indicate the bidentate interaction between R197 and N70A, and the magenta line indicates the lone-pair repulsion between O_distal_ and Oδ70B. (f) 2D representation of the hydrogen-bonding networks in the enolate form (left) and hydroperoxy form (right) chromophores. The bidentate interaction (red dotted lines) and lone-pair repulsion (red schematic lobes) are also indicated.

To further support the presence of a hydroperoxy species in non-photoactivated PAmCherry1, we analyzed the intact protein by high-resolution accurate-mass (HRAM) spectrometry using an Orbitrap mass spectrometer coupled with an electrospray ion source. The deconvolved mass spectrum exhibited two predominant peaks at 28792.3 and 28760.3 Da, corresponding to mass shifts of +12 Da and −20 Da relative to the theoretical mass (Figure 2a). These values are consistent with the hydroperoxy and the enolate forms of the chromophore, respectively. The site of modification was narrowed down to the chromophore via top-down tandem mass spectrometry (MS/MS) analysis using higher-energy collision dissociation (HCD) (Figure S4a,b).

**Figure 2:**
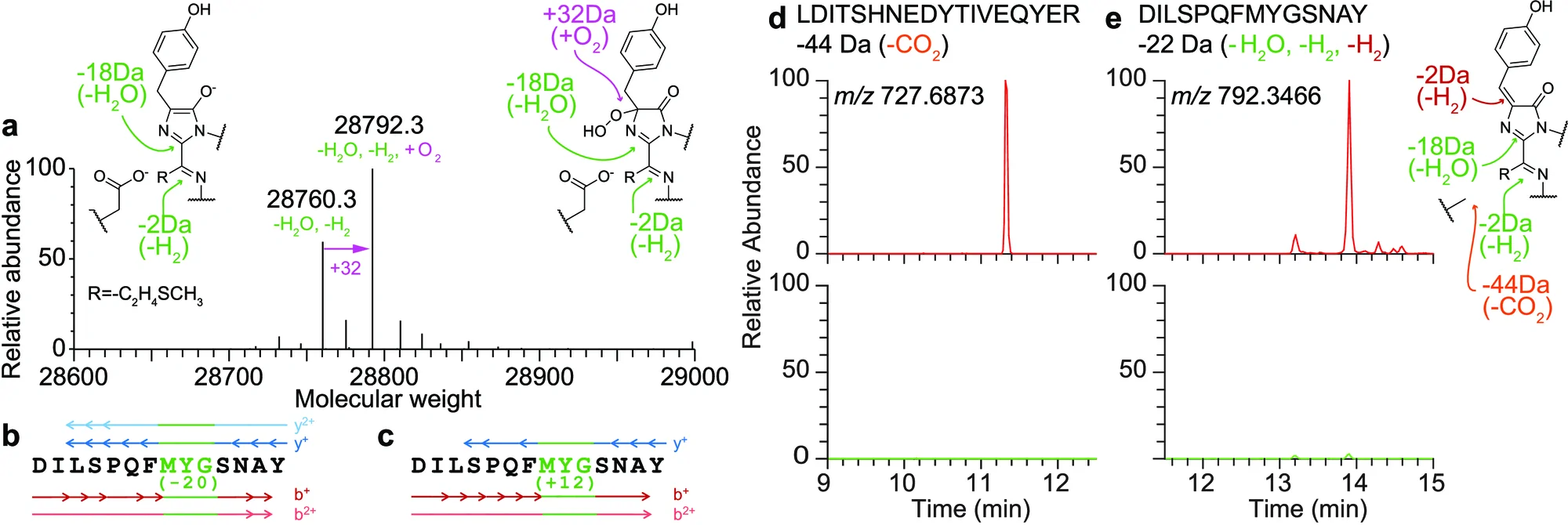
Mass spectrometric characterization of chromophore maturation. (a) Deconvolved HRAM spectrum of non-photoactivated PAmCherry1. Peaks corresponding to +12 Da (−H_2_O, −H_2_, +O_2_) and −20 Da (−H_2_O, −H_2_) mass shifts relative to the theoretical mass are indicated (28792.3 and 28760.3 Da, respectively). Modification sites were further localized by top-down MS/MS analysis (Figure S4). (b, c) MS/MS analyses of chromophore-containing chymotryptic peptides. Arrows on the peptide sequences indicate the observed b- and y-ions derived from precursor ions at *m*/*z* 793.3534 (2+) and *m*/*z* 809.3495 (2+), corresponding to −20 Da and +12 Da species (Figure S4). (d, e) XICs (±10 ppm) of photoactivated PAmCherry1. The tryptic peptide ion containing E215, corresponding to the Kolbe decarboxylation site (*m*/*z* 727.6873 (3+)) (d), and the chymotryptic peptide ion containing the mature chromophore (*m*/*z* 792.3466 (2+)) (e), were extracted. Upper panels show the chromatograms of photoactivated PAmCherry1 (red), whereas bottom panels show the non-photoactivated counterpart (green) as controls. MS spectra and MS/MS analyses of the photoactivated peptides are shown in Figure S5.

This assignment was independently validated through the liquid chromatography-tandem mass spectrometry (LC-MS/MS) analysis of chymotryptic peptides. Two distinct species of chromophore-containing peptides (DILSPQFMYGSNAY) were observed at 13.36 and 13.71 min (Figure S4c,d). The 13.36 min peak showed an ion at *m*/*z* 809.3495 (2+), corresponding to a + 12 Da shift from the theoretical mass, whereas the 13.71 min peak showed an ion at *m*/*z* 793.3534 (2+), corresponding to a −20 Da shift. These masses match the expected values for the two modified species within 2 ppm, strongly supporting their assignment. MS/MS fragmentation further localized the mass shift to the chromophore (Figure 2b,c; Figure S4e,f). Collectively, these results provide mass spectrometric evidence for the coexistence of the hydroperoxy form and enolate form chromophore, consistent with the structural intermediate captured by SFX. Notably, the hydroperoxy species remained detectable through chymotryptic digestion and ESI-MS analysis, indicating unexpected persistence of this intermediate.

Non-photoactivated PAmCherry1 exhibits a single absorption band peaking at 404 nm (Figure S11). To understand this, we performed QM/MM geometry optimizations of the hydroperoxy and enolate chromophore forms (Figure S6) and computed their excited states at the CC2/def2-TZVP level with polarizable embedding. The results indicate that only the enolate form efficiently absorbs 400 nm light (Figure 3), consistent with its more extensive π-conjugation.

**Figure 3:**
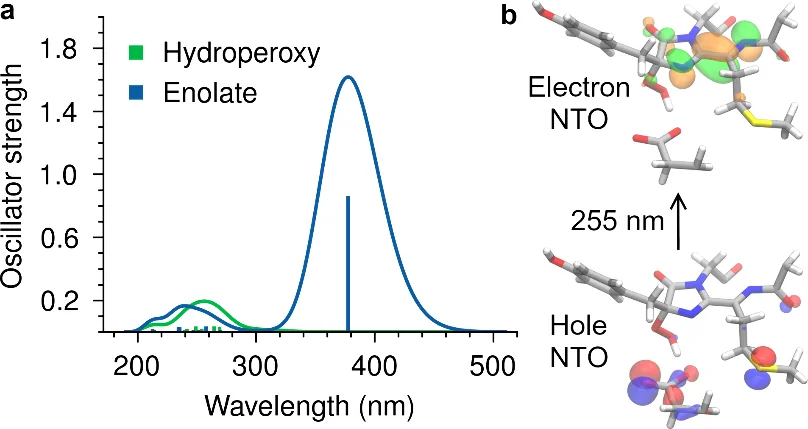
QM/MM calculations of PAmCherry. (a) Absorption spectra of the hydroperoxy and enolate chromophore forms calculated at the CC2/def2-TZVP level with the chromophore in the QM region (states from Tables S5 and S6). (b) Natural transition orbitals for a mechanistically relevant excited state of the hydroperoxy form, with charge-transfer character from E215 to the chromophore (state 7 from Table S7).

Experimentally, the absorbance at 404 nm increased 2.4-fold upon degassing, signifying the enrichment of the enolate form chromophore under anaerobic conditions (Figure 4). This absorbance was fully restored to its initial level after readmitting air. Collectively, these observations allowed us to conclude that the hydroperoxy and enolate form chromophores exist in dynamic equilibrium.

**Figure 4:**
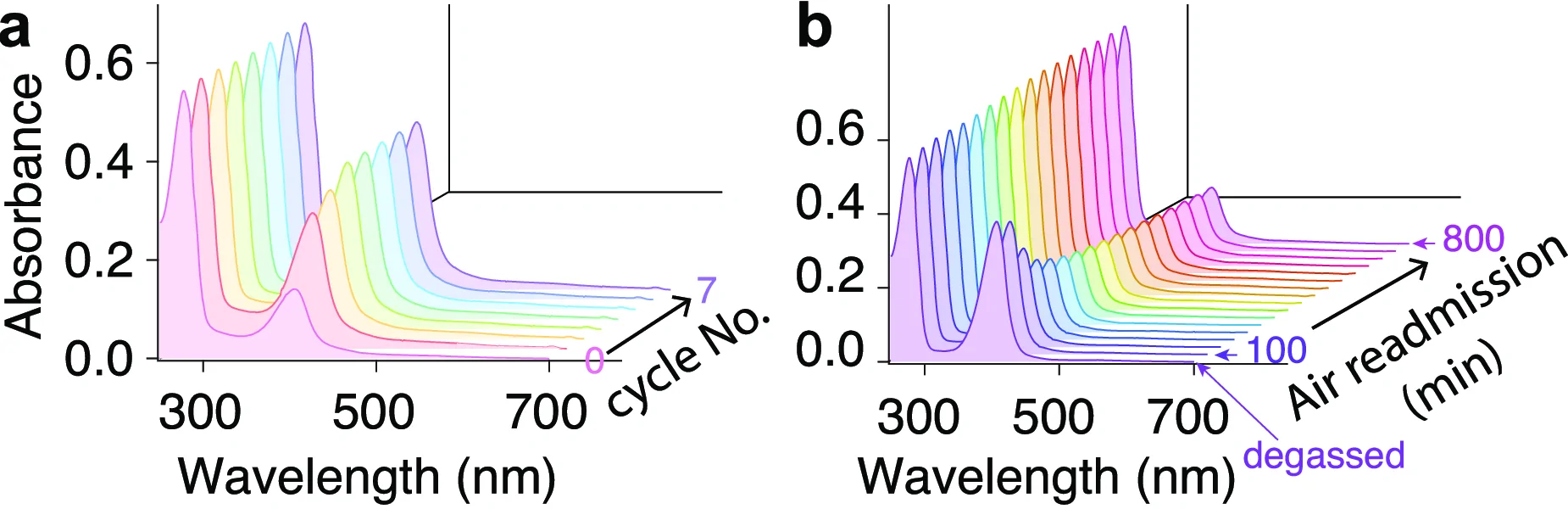
Oxygenation equilibrium shift under rigorous degassing. (a) Sequential freeze–pump–thaw degassing (7 cycles) induced a marked increase in absorbance at 404 nm, which was attributed to a shift in the equilibrium toward the enolate species under anaerobic conditions. (b) Readmission of air triggered the restoration of the hydroperoxy form chromophore, accompanied by a time-dependent decay of the 404 nm absorbance.

The photoactivatable property of PAmCherry1 is conferred by the introduction of mutations at several amino acid positions in mCherry.^8^ Among these residues, N70 and R197 are positioned adjacent to the hydroperoxy moiety, establishing an extensive hydrogen-bonding network together with Q42, E215, E148, and Y181 (Figure 1e,f). This network is likely a key determinant for the stabilization of the hydroperoxy intermediate and the subsequent photoinduced chromophore maturation unique to PAmCherry1. Specifically, a carboxyl oxygen of E215 interacts with the distal oxygen of the hydroperoxy moiety (O_distal_) and the Q42 side chain, while the other carboxyl oxygen interacts with a terminal nitrogen atom of the R197 guanidinium group. The internal nitrogen of R197 forms a hydrogen bond with E148, which in turn communicates with the N70 side chain nitrogen via Y181.

E215 adopts two conformations in the SFX structure. In conformer A, the carboxylate group is not suitably oriented to form a hydrogen bond with O_distal_, exhibiting unfavorable ∠Cδ215A–Oϵ_1_215A···O_distal_ and ∠Oϵ_1_215A···O_distal_–O_proximal_ angles of 76.3° and 69.5°, respectively. In contrast, conformer B adopts a geometry favorable for hydrogen-bond formation to O_distal_ (angles of 112.5° and 100.4°) and simultaneously forms a hydrogen bond with Q42. Thus, only conformer B is compatible with hydrogen-bond formation to the hydroperoxy moiety.

N70 also exhibits two distinct conformations. In conformer A, the carboxamide group engages in a stable bidentate interaction with the R197 guanidinium group, whereas in conformer B, the carboxamide group is rotated away from R197. In the hydroperoxy form, conformer A would place the N70 carboxamide oxygen in close proximity to O_distal_, likely resulting in unfavorable lone-pair repulsion. We therefore propose that N70 adopts the rotated conformer B to alleviate this electronic clash in the hydroperoxy form, while the enolate form is represented by the stable bidentate interaction in conformer A. Conformer B thus represents an energetically strained intermediate, where the hydroperoxy moiety is sequestered despite the electronic repulsion and the loss of the bidentate interaction. This strained arrangement appears to be maintained by E215, whose position is further stabilized through the hydrogen-bonding network involving R197.

To investigate the photoinduced chromophore maturation, we initially attempted to photoconvert PAmCherry1 by illumination with a 400-W xenon lamp through a 400 nm bandpass filter. Despite prolonged exposure for several hours, no significant change in the absorption spectra was observed. In contrast, illumination with a pulsed laser (400 nm, 30 nm fwhm, 60 fs, 150 μW, an average fluence of 0.76 μJ/cm^2^ per pulse) induced the emergence of an absorption peak at 564 nm, a signature of the photoactivated state of PAmCherry1 with a matured chromophore, although the average photon flux of the pulsed laser was 4 orders of magnitude lower than that of the Xe lamp. This conversion was detectable within 5 min and reached a plateau after 25 min of irradiation (Figure S11). Upon excitation of this absorption band at 568 nm, strong red fluorescence peaking at 605 nm was observed (Figure S11). These results suggest that irradiation with 400 nm continuous wave only promotes the protein to the first electronic excited state, whereas illumination with a fs laser may facilitate access to higher-lying electronic states through multiphoton absorption, contributing to photoactivation.

Maturation of the chromophore upon photoactivation was further verified by LC-MS analyses. The decarboxylation of the E215 residue upon photoactivation was confirmed by the observation of a −44 Da mass shift in the tryptic peptide containing the E215 residue (LDITSHNEDYTIVEQY**E**R). In the extracted ion chromatogram (XIC; mass tolerance of ± 10 ppm) of *m*/*z* 727.6873 (3+), a peak corresponding to this mass shift was exclusively detected in photoactivated PAmCherry1 (Figure 2d). Subsequent MS/MS analysis located the mass shift specifically to E215, confirming the photoinduced decarboxylation at this site (Figure S5). The chymotryptic peptide containing the chromophore (DILSPQF**MYG**SNAY) exhibited a −22 Da shift (*m*/*z* 792.3466 (2+)) exclusively in photoactivated PAmCherry1 (Figure 2e). The MS/MS analysis localized this shift to the chromophore-forming region (**MYG**) (Figure S5). Notably, this mass change represents a 34 Da decrease from the hydroperoxy intermediate, a value corresponding to the release of hydrogen peroxide. This observation is consistent with the oxidative dehydrogenation of the chromophore, where the hydroperoxy group acts as an internal oxidant to facilitate Cα67 = Cβ67 double bond formation, thereby completing the maturation of the chromophore.

To directly observe the photoinduced chromophore maturation, we performed a time-resolved SFX (TR-SFX) experiment using a 400 nm fs laser as optical pump laser to evoke the multiphoton photoactivation of PAmCherry1.^14^ The delay time between the pump pulse (a peak fluence of 0.56 J/cm^2^ per pulse, >50 fs) and the XFEL probe pulse was set to 800 ps. The resulting Fourier difference map (*F*_obs_^light‑800 ps^ *-F*_obs_^dark^) between the *light* data set (with pump laser) and the *dark* data set (without pump laser) (Figure 5) showed a clear decrease in electron density around the carboxyl group of E215, which is consistent with the proposed Kolbe decarboxylation mechanism. The negative signal around E215 extends toward the hydroperoxy moiety of the PAmCherry1 chromophore, indicating that its presence is diminishing in concert with the decarboxylation of E215. The disappearance of the hydroperoxy group of the chromophore marks the ongoing Cα67-Cβ67 oxidation to form the mature red chromophore found after photoactivation.

**Figure 5:**
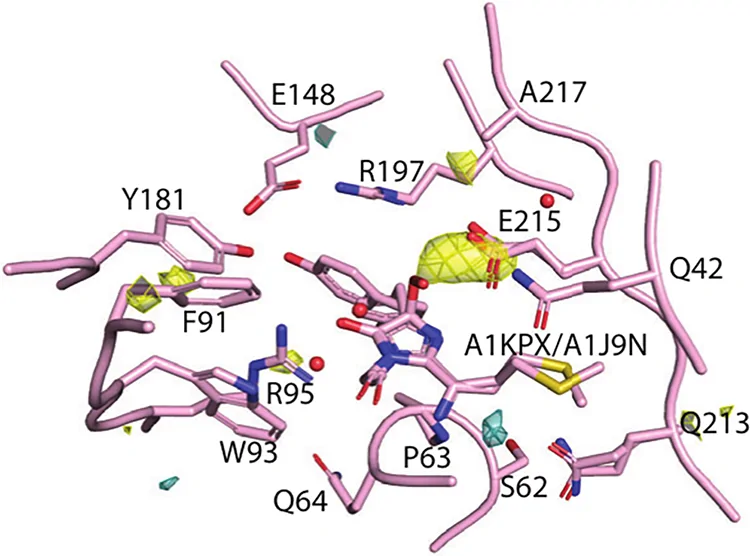
Pump–probe TR-SFX illustrates the PAmCherry1 photoinduced chromophore maturation. *F*_obs_^light‑800 ps^–*F*_obs_^dark^ Fourier difference map (q-weighted) zoomed in on the chromophore pocket of monomer A at 2.50 Å resolution. The oxygen molecule related to the enolate form chromophore is omitted for clarity. A clear decrease in electron density was observed around E215 and the hydroperoxide moiety of the chromophore. (Map contour level: ± 3 r.m.s.d.; teal: positive electron density; yellow: negative electron density; light pink: dark state structure) A general overview of the difference map over the complete asymmetric unit is provided in Figure S13.

To gain further insight into the possible involvement of higher excited states in the photoactivation mechanism, we performed additional QM/MM excited-state calculations at the CC2/def2-TZVP level with a QM region that included residues near the chromophore and analyzed higher excited states of both the hydroperoxy and enolate forms using natural transition orbitals (Figures S9 and S10). For the hydroperoxy form, a state at 255 nm (Figure 3) with small but nonzero oscillator strength (0.01) corresponds to electron transfer from E215 to the chromophore π-system. It could be accessed directly by 250 nm UV excitation or indirectly via an excited state accessible through two-photon absorption at 400 nm. We recently reported that LSSmOrange, a fluorescent protein sharing a common ancestor with PAmCherry1, undergoes photoconversion via Kolbe decarboxylation,^15,16^ with the excited-state chromophore acting as the electron acceptor.^17^ By analogy, the hydroperoxy intermediate state may initiate decarboxylation of E215 during PAmCherry1 photoactivation. No analogous state was found for the enolate form of the chromophore.

Although the precise mechanism of photoinduced maturation remains unresolved, our results support a model in which photoexcitation couples decarboxylation and hydroperoxy elimination. In PAmCherry1, the disruption of the hydrogen-bonding network surrounding E215 as a result of decarboxylation may destabilize the strained N70 conformer B, thereby facilitating the subsequent oxidative maturation process (Scheme 2).

**Scheme 2:**
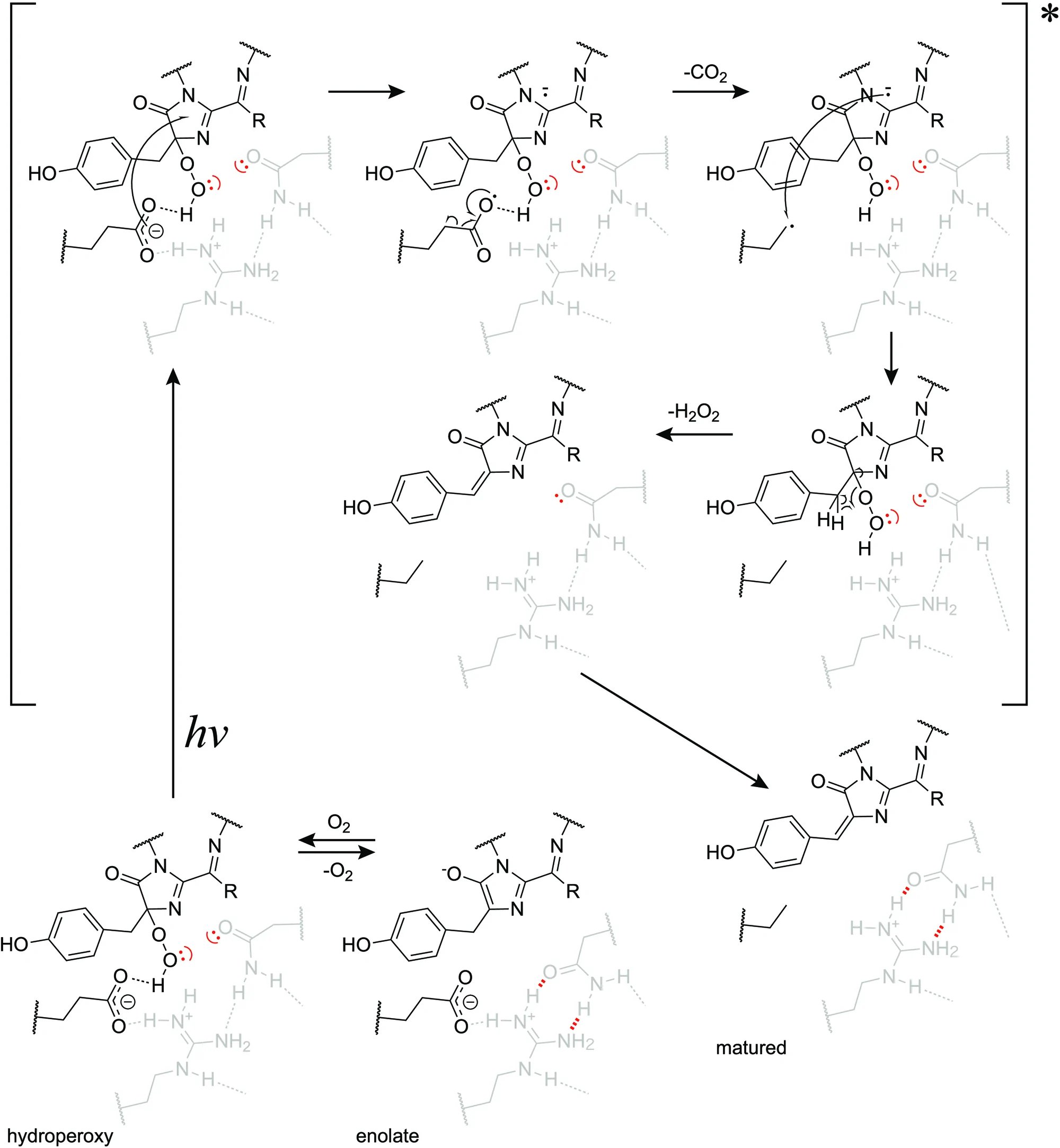
Proposed Photoactivation Mechanism of PAmCherry1

The direct observation of the hydroperoxy intermediate in PAmCherry1 was likely enabled by its stabilization through the unique hydrogen-bonding network. Such a hydrogen-bonding network is absent from other FPs and the hydroperoxy intermediate has therefore not been detected. Nevertheless, because FPs share similar chromophore structures with PAmCherry1, oxidative maturation in other FPs may proceed through a similar mechanism. Whether this mechanism is broadly conserved among fluorescent proteins remains to be established.

In conclusion, we identified a hydroperoxy intermediate in the chromophore maturation of fluorescent proteins in PAmCherry1 using SFX. This elusive species is stabilized by a unique hydrogen-bonding network, while subsequent photoexcitation triggers oxidative maturation of the chromophore. These molecular insights may facilitate the rational design of photoactivatable fluorescent proteins for advanced microscopy.

## Supplementary Material



## Data Availability

The atomic models and structure factors generated in this study have been deposited in the Protein Data Bank (PDB) under accession codes 31AL (synchrotron-based data collected at ESRF ID23-2) and 31JT (SFX-based data collected at SACLA BL2­(EH3)). Additional PDB entries referenced in this work are publicly accessible through the PDB repository. The CIF files containing the definitions and restraints for the enolate form and hydroperoxy form chromophores are available within the PDB depositions. Raw diffraction images supporting these findings have been archived in the Coherent X-ray Imaging Data Bank (CXIDB) under entry ID 243.

## Notes

Materials and methods; synchrotron, SFX, and time-resolved SFX crystallographic analyses, including crystallographic and refinement statistics; high-resolution accurate-mass spectrometric analyses; QM/MM structure optimization and CC2 excited-state calculations; free-energy calculations; and femtosecond-laser photoactivation experiments (PDF)

## ABBREVIATIONS

ESI-MS: electrospray ionization mass spectrometry
FP: fluorescent protein
HCD: higher-energy collision dissociation
HRAM: high-resolution accurate-mass
LC-MS/MS: liquid chromatograph-tandem mass spectrometry
MS/MS: tandem mass spectrometry
SFX: serial femtosecond crystallography
TR-SFX: time-resolved serial femtosecond crystallography
XFEL: X-ray free-electron laser
XIC: extracted ion chromatogram
