# Supplementary material for: Direct Visualization of a Hydroperoxy Intermediate in Fluorescent Protein Chromophore Maturation in PAmCherry1
Source: J Am Chem Soc. 2026 Sep 21;148(38):40622–8. doi: 10.1021/jacs.6c13605 (PMC13632553; doi:10.1021/jacs.6c13605)
Supplement: Supplementary file 1 [file ja6c13605_si_001.pdf]

Supporting information

Direct visualization of hydroperoxy intermediate in fluorescent protein chromophore maturation in PAmCherry1

Hanifa Bibi[‡a], Leonie De Vrieze[‡a], Elke De Zitter[b], Nicolas Coquelle[b], Janko Civic[c], Eduard Fron[d], Kenichi Kamata[e], Masaya Usui[f], Kaori Otsuki[f], Gilles Garré[a], Ronald Rios-Santacruz[b], Giorgio Schirò[b], Fangjia Luo[g], Nipawan Nuemket[g,h], Jungmin Kang[h], Shigeki Owada[g,h], Kensuke Tono[g,h], Thi Yen Hang Bui[i], Olivia Olislaegers[a], Hidetsugu Asada[j], Dohyun Im[j], Luc Van Meervelt[k], So Iwata[j], Nobuhiko Miyasaka[f], Jeremy N. Harvey[c], Eriko Nango[l], Martin Weik[b], Hideaki Mizuno[*a]

a. Laboratory of Biomolecular Network Dynamics, Biochemistry, Molecular and Structural Biology Section, Department of Chemistry, KU Leuven, Celestijnenlaan 200G bus 2403, 3001 Leuven, Belgium

b. Univ. Grenoble Alpes, CEA, CNRS, Institut de Biologie Structurale, 38000 Grenoble, France

c. Quantum Chemistry and Physical Chemistry Section, Department of Chemistry, KU Leuven, Celestijnenlaan 200F bus 2404, 3001 Leuven, Belgium

d. Chem&Tech-Molecular Imaging and Photonics, Department of Chemistry, KU Leuven, Celestijnenlaan 200F bus 2404, 3001 Leuven, Belgium

e. College of Science and Technology, Nihon University, 1-5-1 Kandasurugadai, Chiyoda-ku, Tokyo 101-0062, Japan

f. Support Unit for Bio-Material Analysis, RIKEN Center for Brain Science, 2-1 Hirosawa, Wako-city, Saitama 351-0198, Japan

g. Japan Synchrotron Radiation Research Institute, 1-1-1 Kouto, Sayo, Hyogo 679-5198, Japan

h. RIKEN SPring-8 Center, 1-1-1 Kouto, Sayo-cho, Sayo-gun, Hyogo, 679-5148, Japan

i. Faculty of Chemistry, Hanoi National University of Education, 136 Xuan Thuy street, Cau giay district, Hanoi, Vietnam

j. Department of Cell Biology, Graduate School of Medicine, Kyoto University, Yoshidakonoe-cho, Sakyo-ku, Kyoto 606-8501, Japan

k. Laboratory of Biomolecular Architecture, Biochemistry, Molecular and Structural Biology Section, Department of Chemistry, KU Leuven, Celestijnenlaan 200G bus 2403, 3001 Leuven, Belgium

l. Institute of Multidisciplinary Research for Advanced Materials, Tohoku University, Aoba-ku, Sendai 980-8577, Japan

[‡] These authors contributed equally
[*] Corresponding author

Table of contents

Supplementary Text 1: Structural analyses of non-photoactivated PAmCherry1

Supplementary Text 2: Free-energy difference between the hydroperoxy and enolate chromophore forms

Materials and methods

Supporting Tables

Table S1. Crystallographic analysis and refinement statistics for PAmCherry1

Table S2. Equilibration protocol used to generate QM/MM optimized structures of PAmCherry1

Table S3. Composition of the QM region for each PAmCherry1 form during QM/MM structure optimization. Residue numbering follows the original PDB files

Table S4. Composition of the QM region for each PAmCherry1 form for QM/MM excited-state calculations. Residue numbering follows the original PDB files

Table S5. Vertical excitation energies and oscillator strengths of the QM/MM-optimized hydroperoxy form of PAmCherry1, calculated for the small QM region (Table S4) at the CC2/def2-TZVP level

Table S6. Vertical excitation energies and oscillator strengths of the QM/MM-optimized enolate form of PAmCherry1, calculated for the small QM region (Table S4) at the CC2/def2-TZVP level

Table S7. Vertical excitation energies and oscillator strengths of the QM/MM-optimized hydroperoxy form of PAmCherry1, calculated for the big QM region (Table S4) at the CC2/def2-TZVP level with the RVS approximation

Table S8. Vertical excitation energies and oscillator strengths of the QM/MM-optimized enolate form of PAmCherry1, calculated for the big QM region (Table S4) at the CC2/def2-TZVP level with the RVS approximation

Supporting Figures

Figure S1. PAmCherry1 crystals used for X-ray crystallography

Figure S2. Synchrotron radiation structure of non-photoactivated PAmCherry1

Figure S3. Crystal structure of non-photoactivated PAmCherry1 solved by SFX

Figure S4. MS analysis of the non-photoactivated PAmCherry1

Figure S5. LC-MS analysis of the photoactivated PAmCherry1

Figure S6. Comparison of QM/MM optimized and crystal structures of PAmCherry1 in the hydroperoxy and enolate forms

Figure S7. Most significant hole and electron natural transition orbitals (NTOs) of PAmCherry1 hydroperoxy form excited states, calculated with CC2/def2-TZVP

Figure S8. Most significant hole and electron natural transition orbitals (NTOs) of PAmCherry1 enolate form excited states, calculated with CC2/def2-TZVP

Figure S9. Most significant hole and electron natural transition orbitals (NTOs) of PAmCherry1 hydroperoxy form excited states, calculated with RVS-CC2/def2-TZVP and a big QM region

Figure S10. Most significant hole and electron natural transition orbitals (NTOs) of PAmCherry1 enolate form excited states, calculated with RVS-CC2/def2-TZVP and a big QM region

Figure S11. PAmCherry1 photoactivation by illumination with a fs-pulse laser

Figure S12. No differences were observed between the SFX dataset of non-photoactivated PAmCherry1 (complete dark) and the interleaved dark dataset of the TR-SFX experiment

Figure S13. Pump-probe TR-SFX illustrates the PAmCherry1 photoinduced chromophore maturation in monomer A of the model

Figure S14. Thermodynamic cycle used to estimate the relative free energies of the hydroperoxy and enolate forms of the PAmCherry chromophore

References

Supplementary Text 1: Structural analyses of non-photoactivated PAmCherry1

Synchrotron radiation structure

The structure was solved by molecular replacement using a previously determined structure (PDB ID:3KCS) as search model. The crystals diffracted to 1.5 Å and belonged to the orthorhombic space group $P22_12_1$ with two molecules in asymmetric unit (monomer A and monomer B). The final R_{work} and R_{free} values were 17.87% and 22.17%, respectively. The overall structure of PAmCherry1 was a typical 11-stranded β-barrel fold surrounding the chromophore, which is composed of amino acid residues M66-Y67-G68 and fits to 4-acylimine-2-(4-hydroxybenzyl)imidazolinone (Figure S2 a-c).

The structure was consistent with the previously reported structure (RMSD of 0.227 Å and 0.197 Å for 197 Cα atoms in monomers A and B, respectively), with a variation in the conformation of the chromophore hydroxybenzyl moiety. The conformation in monomer B is consistent with the reported structure of non-photoactivated PAmCherry1 (3KCS), whereas that in monomer A exhibits two conformers. The minor conformer (conformer A, occupancy of 47%) matches the conformation observed in monomer B. Although the major conformer (occupancy of 53%, conformer B) resembles that of 3KCT, reported as the photoactivated state, we do not attribute this to photoactivation, as the chromophore remains in the enolate form without formation of the Cα67=Cβ67 double bond, as described below.

For all chromophore conformations present in the structure, the Cα67-Cβ67 bond is nearly coplanar with the imidazolinone ring, as indicated by a small deviation angle of 1~2°, signifying an sp^2-like geometry at Cα67 (Figure S2d). Together with the nearly coplanar geometry, the Cα67-C67 (1.35~1.37 Å) and C67-O67 (1.38~1.39 Å) bond lengths are consistent with the enolate form imidazolinone tautomerism. The side chain of R95 stabilized the anionic oxygen of the enolate form chromophore (Figure S2b,d). The distance between the anionic oxygen (O67) of the imidazolinone ring and a terminal nitrogen atom of the guanidinium moiety is 2.7~2.9 Å with a C–O⋯N angle of ~140°, indicating the formation of a hydrogen bond. The Cα67−Cβ67 bond length of 1.53 Å, combined with the 33.2° (major conformer monomer A) or 60.5~63.8° (minor conformer monomer A, and monomer B) orientation of the Cβ67−Cγ67 bond with the imidazolinone plane indicates sp^3 hybridization at Cβ67. This geometry is incompatible with formation of a Cα67=Cβ67 double bond, which would be required to conjugate the two rings.

SFX structure

As described in the main text, the best-fit model for the chromophore in monomer A was obtained using a two-state model comprising an enolate form chromophore with modeled molecular oxygen and a hydroperoxy form chromophore with refined occupancies of 27% and 73%, respectively (Figure 1d, S3b). Similarly, we first refined the chromophore structure of monomer B using the enolate form model (Figure S3c). The conformation of the hydroxybenzyl moiety is consistent with those observed in monomer B and in the minor conformer of monomer A in the synchrotron structure. However, significant negative mF_{obs}-DF_{cal} electron density appears on one side of the benzyl moiety, with a corresponding positive peak on the opposite side, suggesting an alternative conformation. In addition, as observed for monomer A, a prominent positive electron density peak is found at Cα67, indicative of a tetrahedral geometry. We therefore refined the structure further using a hydroperoxy form model (Figure S3c). In this model, the benzyl moiety adopts a conformation consistent with the alternative conformation suggested by the enolate model. This conformation is similar to the hydroperoxy form chromophore in the two-state model of monomer A and the major conformation in monomer A of the synchrotron model. Residual positive and negative mF_{obs}-DF_{cal} electron density around the benzyl moiety in the hydroperoxy form model indicates a partial contribution of the enolate form. Finally, a two-state model comprising an enolate form chromophore with modeled molecular oxygen and a hydroperoxy form chromophore was refined to occupancies of 51% and 49%, respectively (Figure S3c). During the peer-review process, the possibility that this electron density could instead be attributed to a dynamic water molecule was raised. We therefore refined the two-state model with a water molecule instead of molecular oxygen. This refinement left residual positive mF_{obs}-DF_{cal} electron density, whereas molecular oxygen satisfactorily accounted for the observed density (Figure S3c).

In short, both hydroperoxy and enolate chromophore forms were observed in monomer A and monomer B of the asymmetric unit, but the occupancy ratio of both chromophore species was different. In monomer A, the hydroperoxy form chromophore was predominant while the ratio in monomer B was more balanced.

Radiation damage-free structure in SFX

In the synchrotron structure, significantly reduced electron density was observed in the $2mF_{obs}$-DF_{cal} map at the E215 side chain (Figure S2e). This could be attributed to X-ray radiation damage (absorbed X-ray dose for the data set was 11.58 MGy). In addition, no significant radiation damage was observed for other glutamate and aspartate residues in the structure. This high sensitivity of E215 to X-ray radiation may arise from its proximity to the nucleophilic chromophore; the distance between $O\varepsilon_2$215 and $N_2$67 in the chromophore is 2.8~3.0 Å. Similar radiation sensitivity has been reported for

LSSmOrange[1]. In the SFX structure, intact electron density is observed around the entire side chain of E215, indicating that the PAmCherry1 structure was obtained without significant radiation damage by SFX, consistent with the principle of 'diffraction before destruction' (Figure S3d).

N70 conformations

N70 and R197 were introduced to confer the photoactivatable property to PAmCherry1. N70 adopts two conformations (conformers A and B) (Figure S3e). In conformer A, the carboxamide group (Oδ70A, Nδ70A) forms bidentate interactions with the terminal nitrogen atoms of the R197 guanidinium group (N$\eta_1$197, N$\eta_2$197) with distances of 2.8~2.9 Å and 3.0~3.2 Å, respectively. These bidentate interactions are disrupted in conformer B. We propose that the hydroperoxy form favors conformer B because the disruption of the bidentate network relieves the lone-pair repulsion between Oδ70 and O_{distal}. In conformer A, the distance between Oδ70 and O_{distal} is only 3.4~3.6 Å, whereas it increases to 4.8~4.9 Å in conformer B. In contrast, the enolate form lacks the O_{distal} atom responsible for this repulsion and therefore preferentially adopts conformer A, which is stabilized by the bidentate interactions.

E215 conformations

E215 adopts two conformations in the SFX structure (Figure S3d). Conformer A (occupancy of 27~51 %) is conformation incompatible with hydrogen-bond formation to O_{distal} of the hydroperoxy group, as indicated by the unfavorable ∠Cδ215A–O$\varepsilon_1$215A···O_{distal} angle of 73.3~82.1° and ∠ O$\varepsilon_1$215A···O_{distal}–$O_{proximal}$ angle of 69.5~75.8°. In contrast, in conformer B (occupancy of 49~73 %), O$\varepsilon_1$215B adopts a geometry suitable for hydrogen-bond formation with O_{distal}, with O$\varepsilon_1$215B··· O_{distal} distance of 2.4 Å, ∠Cδ215B–O$\varepsilon_1$215B···O_{distal} angle of 112.4~112.5°, and ∠ O$\varepsilon_1$215B··· O_{distal}–$O_{proximal}$ angle of 100.4~102.3°. O$\varepsilon_1$215B also forms a hydrogen bond with the carboxamide nitrogen atom of Q42 at a distance of 3.1~3.2 Å. We therefore propose that E215 adopts conformer B in the hydroperoxy form chromophore, where it forms hydrogen bonds with both O_{distal} and Q42, whereas conformer A corresponds to the enolate form chromophore in which O_{distal} is absent.

Supplementary Text 2: Free-energy difference between the hydroperoxy and enolate chromophore forms

To assess whether the proposed equilibrium between the hydroperoxy and enolate forms of the non-photoactivated PAmCherry chromophore is not energetically unreasonable, we computed a rough estimate of their relative free energies using truncated chromophore models. Because the two forms differ by an O_2 unit and a proton, the thermodynamic cycle also involves molecular oxygen and a proton source, modelled here as an isolated acetic acid/acetate species (Figure S14).

Geometries of all isolated species (the two chromophore models, O_2, and acetic acid) were optimized at the B3LYP/def2-TZVP level of theory with the CPCM implicit solvent model using parameters for chloroform, chosen to approximate the dielectric environment inside a protein, followed by frequency calculations to obtain thermodynamic corrections at 298.15 K, including the zero-point vibrational energy.

The hydroperoxy form was computed to lie 6.7 kcal/mol higher in free energy than the enolate + O_2 reference state. This favors the enolate form, but the protein environment is completely absent from this minimal model. Specific interactions within the chromophore pocket (for example, hydrogen bonding to the hydroperoxide group, or electrostatic stabilization from nearby residues) could possibly shift the balance by several kcal/mol and stabilize the hydroperoxy form sufficiently for the two forms to coexist in equilibrium. The calculation is intended only to show that the proposed equilibrium is energetically plausible; a quantitative estimate would require QM/MM free-energy simulations in the protein environment.

Materials and Methods

Cloning, protein expression and purification of recombinant PAmCherry1

The PAmCherry1 coding sequence was PCR amplified using the pTfR-PAmCherry1 plasmid (a gift from Vladislav Verkhusha; Addgene plasmid #31948; http://n2t.net/addgene:31948 ; RRID:Addgene_31948) as a template.[2] To obtain the C-terminal His6-tagged (C-His PAmCherry1) construct used for crystallization, the PAmCherry1 gene was inserted between the NcoI and XhoI sites in the pET28 vector. The ATG codon in the NcoI recognition site served as the start codon of the protein. The PAmCherry1 sequence was linked to the His6-tag encoded in pET28 by the in-frame addition of the recognition site (6 bp) of XhoI. The XhoI site added a leucine-glutamate linker between PAmCherry1 and the tag. The N-terminal His6-tagged construct of PAmCherry1 (N-His PAmCherry1) used for mass spectrometry analysis was the result of the insertion of the PAmCherry1 gene between the NdeI and XhoI sites in the pET28c expression vector. The ochre stop codon (TAA) was added at the end of the PAmCherry1 sequence, before the XhoI site. The NdeI recognition sequence was added in front of the PAmCherry1 sequence, overlapping with the ATG start codon. This yielded a vector encoding PAmCherry1 in frame with the N-terminal His6-tag and thrombin cleavage site included in the pET28c sequence.

C-His PAmCherry1 and N-His PAmCherry1 were expressed in *Escherichia coli* JM109(DE3) (Promega) transformed with the corresponding expression vectors using kanamycin (50 µg/mL) as a selection marker. A single colony was picked from an LB agar plate and grown for 6 hours in 1 mL of LB medium. This small culture was inoculated in 2 L (C-His PAmCherry1) or 1 L (N-His PAmCherry1) of LB medium and grown overnight at 21°C till an OD_{600} of 0.1-0.2 was reached. Expression was induced with 100 µM isopropyl β-D-thiogalactoside (IPTG) and culture growth was continued for 24 hours ($OD_{600} > 1$). The cells were harvested by centrifugation, resuspended in phosphate-buffered saline (PBS) and frozen. After thawing the frozen suspension, 50 µM phenylmethylsulfonyl fluoride (PMSF), 0.1 mg/mL lysozyme, 1 mM $MgCl_2$ and 10 µg/mL DNase I were supplemented. After three additional freeze-thaw cycles, the cell debris was removed by centrifugation. The resulting supernatant was subjected to metal affinity chromatography with Ni-NTA agarose beads (Qiagen). The proteins were eluted with 50 mM Tris-HCl buffer pH 7.5 containing 300 mM NaCl and 200 mM imidazole. Size exclusion chromatography was applied for further purification. A HiLoad 16/600 Superdex 75 preparative grade column (Cytiva) on an Äkta system was used with 20 mM HEPES pH 7.5, 150 mM NaCl as running buffer. The purity of the proteins was evaluated by SDS-PAGE. Purified recombinant PAmCherry1 was concentrated by ultrafiltration using a Vivaspin Turbo 15 filtering device (molecular weight cut-off of 10,000 Da; Sartorius). To prepare C-His PAmCherry1 for crystallization, the buffer was exchanged for 10 mM HEPES pH 7.5 using a PD-10 desalting column (Cytiva). Protein concentration was estimated based on the absorbance at 404 nm using the reported extinction coefficient 6,500 M^{-1} cm^{-1}.[2]

Crystallization

PAmCherry1 crystals for synchrotron diffraction data collection were grown using the hanging drop vapor diffusion method. Crystals were prepared by mixing 1 µL protein solution (15 mg/mL) and 1 µL precipitant solution (0.2 M sodium acetate, 20% (w/v) PEG 3350). The resulting drop was equilibrated against a reservoir of precipitant solution at 20°C. Crystals (100 µm x 95 µm x 42 µm) grew in the drops within 3-4 weeks (Figure S1a).

Microcrystals for SFX diffraction data collection were grown using the batch crystallization method combined with microseeding. The initial seeds were created using the crystals obtained by the hanging drop vapor diffusion method. The crystals were harvested together with their mother liquor and crushed by vortexing the crystals with seed beads (Hampton Research) in 0.2 M sodium acetate, 20% (w/v) PEG 3350. Fresh crystals were grown on 96-well microbatch plates (Swissci) which were sealed with transparent tape. The drops were composed of 5 µL precipitant solution (0.2 M sodium acetate, 28-30% (w/v) PEG 3350), 5 µL protein solution (25 mg/mL) and 0.25 µL seed solution. Smaller sized crystals (ca. 30-50 µm) grew overnight at 20°C (Figure S1b). These crystals were used to make fresh seeds for the larger scale batch crystallization of PAmCherry1. The drops with the smaller-sized crystals were harvested and diluted in 0.2 M sodium acetate, 20% (w/v) PEG 3350. The crystals were crushed using a BeadBug D1030 microtube homogenizer (Benchmark Scientific) and a combination of multiple TriplePure High Impact Zirconium beads of 3.0 mm and 1.5 mm (Benchmark Scientific). The microcrystals used for SFX measurements were grown in a batch size of 550 µL in 1.5 mL low protein binding microcentrifuge tubes (Thermo Fisher). Protein solution (25 mg/mL) was mixed in a 1:1 ratio with precipitant solution (0.2 M sodium acetate, 28-32% (w/v) PEG 3350) and 50 µL of seed solution was added. Crystals with a suitable size (5-30 µm) were obtained after overnight incubation at 20 °C (Figure S1c).

Synchrotron diffraction data acquisition

Single crystals were harvested in their mother liquor using a cryo-loop and were flash frozen in liquid nitrogen. Diffraction data was collected at the ID23-2 beamline of the European Synchrotron Radiation Facility (ESRF) on a Dectris Eiger 2 9M detector at 100K.[3] An X-ray beam with a wavelength of 0.8731 Å was used which had a spot size of 4 × 25 µm². The total oscillation range was 360° (0.1° oscillations per frame; 3600 frames collected). Indexing and integration of the diffraction spots were done using XDS. Aimless was used to scale the data, to add R_{free} flags (5% of the reflections) and to cut the dataset at 1.50 Å resolution.[4] RADDOSE-3D was used to calculate the absorbed X-ray dose for the data set. [5]

SFX and pump-probe TR-SFX diffraction data collection

SFX experiments were performed at the BL2(EH3) XFEL beamline of the Spring-8 Angstrom Compact free electron LAser (SACLA) using the DAPHNIS platform with a closed sample chamber.[6,7] The XFEL pulses (pulse duration of 10 fs) had a photon energy of 10 keV, were focused to a spot size of 1.5 µm at full width at half maximum (FWHM) and were directed on the microcrystals with a repetition rate of 30 Hz. Diffraction patterns were collected on a MPCCD-phase III detector at a sample-to-detector distance of 50 mm.[8] For the pump-probe TR-SFX experiments, right circularly polarized femtosecond pump pulses provided by SACLA synchronized femtosecond laser system were used at a repetition rate of 15 Hz to collect diffraction data in a light-dark regime. The 401-nm pulses of 12.5 µJ had a pulse duration of >50 fs and were focused to an illumination area of 29.2 µm x 34.0 µm (FWHM). The pulse energy was reduced by 50% upon conversion to circular polarization. Assuming a Gaussian spatial profile, the effective peak laser fluence was calculated to be 0.56 J/cm^2 per pulse as follows:

$$F_{peak} = E\frac{ln(2)}{A_{FWHM}}$$

where A_{FWHM} is the area defined by the FWHM dimensions. The beam center of the pump pulses was aligned to the beam center of the XFEL. Three data sets were collected: SFX of non-photoactivated PAmCherry1 (no pump laser), TR-SFX dark interleaved (no pump laser) and TR-SFX light-800 ps (800 ps delay between pump laser and XFEL pulse).

A high-viscosity cartridge-type injector (nozzle inner diameter of 75 µm) was used to deliver the microcrystals to the XFEL interaction site.[9,10] The cartridge (total volume: 200 µL) was loaded with a mixture of 20-40 µL sedimented microcrystal slurry with 160-180 µL 20% hydroxyethyl cellulose (containing 0.1 M sodium acetate and 15% (w/v) PEG 3350). The flow rate of the injector was 2.4 µL/min. The chilling system for the high-viscosity injector was set at 20°C.

The Cheetah pipeline software at SACLA was used to extract "hit" images.[11] Sample-to-detector distance (clen) was optimized by varying the clen value and selecting the one that resulted in the most indexed patterns and the most symmetrical unit cell histograms as visualized by Unit Cell Explorer included in CrystFEL.[12] Further detector geometry optimization was performed by detector-shift (CrystFEL). The recorded images were indexed by indexamajig (CrystFEL) (unit cell pre-defined based on synchrotron structure) using XGANDALF[13], Mosflm[14] and DirAx.[15] The peaks were detected by the Peakfinder8 algorithm with a threshold of 1200, a minimal signal-to-noise ratio of 3.5 and an integration radius of (3,4,7). The tolerance was 10% for the a and c axis of the unit cell, 2% for the b axis and 2.5° for the unit cell angles. The "multi" function was enabled to index multiple lattices in a single diffraction image. The other parameters were kept default. The data were merged using the Monte Carlo method implemented in process_*hkl* within CrystFEL. The resolution cut-off was determined based on the outer shell statistics. The resolution of the SFX (complete dark) dataset was 1.63 Å while the resolutions of the interleaved dark dataset and the light dataset were 1.57 Å and 1.68 Å, respectively. Data truncation was done by truncate.[16] R_{free} flags were added using the "uniqueify" script.

Structure solution, refinement and calculation of Fourier difference maps

The initial phases of the synchrotron model of PAmCherry1 were obtained by molecular replacement using Phaser MR and PDB ID:3KCS as search model. For the SFX (complete dark) dataset and the interleaved dark dataset, the phases of our synchrotron model were used as initial estimates. The model of PAmCherry1 was built in COOT[17] and refined using phenix.refine (Phenix).[18] CIF files containing the restraints for the different chromophore species were created using phenix.elbow followed by manual inspection[19]. A1KPX was used for the enolate form chromophore, while A1J9N represents the hydroperoxy form chromophore. The chromophores were manually linked to the neighboring amino acid residues using restraints based on the trans peptide bond. The refinement statistics are shown in Table S1.

Fourier difference maps were calculated by Xtrapol8 (version 1.2.8) using a q-weighting scheme.[20–22] Other parameters were kept default. Before calculation of the Fourier difference maps both light and interleaved dark datasets were cut to 2.5 Å resolution. The resulting datasets were highly isomorphous, as assessed by the overall R_{iso} value (0.07). There were no significant differences between the complete dark SFX data set and the interleaved dark dataset (Figure S12).

Mass spectrometry analysis

All mass spectrometric analyses were performed using the Q Exactive Orbitrap mass spectrometer (Thermo Fisher Scientific). For intact protein mass measurements and top-down analyses, protein samples were buffer-exchanged into 10 mM ammonium acetate with gel filtration spin columns (Micro Bio-Spin P-6 Gel Columns; Bio-Rad) and adjusted to a final concentration of 100 µM. Samples were introduced into the mass spectrometer by positive-mode electrospray ionization using the TriVersa NanoMate (Advion) at 1.8 kV with a 5-µm inner-diameter ESI chip. Mass spectra were acquired over 30 s from *m/z* 1000 to 4500 with a resolution of 140,000 (FWHM) at *m/z* 200. Spectra averaged over the acquisition period were deconvoluted using Xcalibur (Thermo Fisher Scientific) to determine the monoisotopic mass of the intact protein. For top-down MS/MS analysis, ions at 15+ charge states were selected as precursors and fragmented using higher-energy collisional dissociation (HCD) with normalized collision energies (NCE) ranging from 10% to 30%. The resulting spectra were analyzed using BioPharma Finder (Thermo Fisher Scientific) for the assignment of b- and y-type ions.

For LC–MS/MS peptide analyses, proteins were first subjected to enzymatic digestion.[23] Briefly, 5 µL of protein solution was mixed with 15 µL of 6 M guanidine hydrochloride and incubated at 80 °C for 3 min. After cooling to room temperature, 100 µL of 50 mM ammonium bicarbonate was added, and the solution was divided into two equal aliquots. One aliquot was digested with chymotrypsin (3.2 ng/µL) at 25 °C for 15 h, while the other was digested with trypsin (3.2 ng/µL) at 37 °C for 15 h. Peptides were desalted with an in-house C18 StageTip prior to analysis. LC–MS/MS measurements were carried out using the Vanquish Neo UHPLC system (Thermo Fisher Scientific) coupled online to the Q Exactive mass spectrometer via a nanospray ion source. Peptides were separated on a NANO-HPLC capillary column (C18, 75 µm × 150 mm, 3 µm particle size; Nikkyo Technos) with a 20 min gradient at a flow rate of 300 nL/min (5–35% solvent B over 10 min, 35–95% over 4 min, followed by 95% for 6 min; solvent A: 0.1% formic acid in water; solvent B: 0.1% formic acid in acetonitrile). MS and MS/MS data were searched against a custom sequence database containing PAmCherry1 using Proteome Discoverer (version 3.1; Thermo Fisher Scientific) with the Mascot search engine (version 3.1; Matrix Science). Extracted ion chromatograms (XICs) were generated with a mass tolerance of 10 ppm using Xcalibur.

QM/MM computational details

For the protein in the dark state, both the hydroperoxy and the previously proposed enolate forms were considered. The starting structure for the hydroperoxy form was taken from this work, whereas the structure for the enolate form was obtained from PDB ID 3KCS.[24] Crystal water molecules within 8 Å of the chromophore were retained. For residues with alternate conformations, the major occupancy state was selected. Missing heavy atoms and hydrogen atoms were added using the reduce program from AmberTools23.[25] The protonation states of residues near the chromophore were manually inspected. In both models, E215 was considered deprotonated.

The protein was solvated with 0.15 M NaCl in an octahedral box ensuring a minimum distance of 12 Å between the protein and the box boundaries. The ff19SB[26] force field was used for the protein, and TIP3P[27] for water molecules. The force field parameters for the chromophore residue were developed in the same way as described in our previous work.[28]

The structures were optimized using a multistep protocol (Table S2), starting with classical MD relaxation while restraining the chromophore region, followed by unconstrained QM/MM optimization. The QM regions, comprising approximately one hundred atoms (Table S3), were treated with the composite PBEh-3c method[29], while the remainder of the system was described classically using point charges from the force field. All calculations were carried out with Amber22[30–34] and ORCA 5.[35]

Using the optimized structures, excited-state QM/MM calculations were performed at the CC2/def2-TZVP level employing the resolution-of-the-identity (RI) approximation. The QM region included the chromophore residue capped with acetyl and methylamine groups, while the remainder of the system was described using polarizable embedding, with parameters developed as in our previous work[28] using the PyFraME package[36]. Fifteen excited states were computed for each structure (Tables S5, S6). Natural transition orbitals of each state are shown in Figures S7 and S8. The calculated stick spectra were broadened using a sum over all excited states i with energy E_i and oscillator strength f_i:

$$S(E) = \sum_{\mathrm{i}} f_{\mathrm{i}} \frac{1}{\sigma\sqrt{2\pi}} \exp\left[-\frac{(E-E_{\mathrm{i}})^2}{2\sigma^2}\right]$$
$$\sigma = \frac{0.5\ \mathrm{eV}}{2\sqrt{2\ln(2)}}$$

To search for higher-lying excited states that could plausibly be involved in the photoactivation mechanism additional CC2/def2-TZVP excited-state calculations were performed with a big QM region that included residues in the vicinity of the chromophore (Table S4). These employed the restricted virtual space approximation, with virtual orbitals above 60 eV excluded from the correlated equations; the surrounding system was again described with polarizable embedding. We have

previously shown this approach to reliably reproduce charge-transfer excitation energies.[28] Ten excited states were computed for each structure (Tables S7, S8). Natural transition orbitals of each state are shown in Figures S9 and S10. All excited-state calculations were carried out with Turbomole.[37–40]

Degassing of PAmCherry1 solution

Degassing was performed by freeze-pump-thaw cycles. We designed a T-shaped custom-made anaerobic cell (T-cell) consisting of a 1-cm path length quartz cuvette vertically connected to a sample reservoir, and a high-vacuum valve equipped with a port for attachment to a vacuum line. The protein solution was frozen in liquid nitrogen. The T-cell was evacuated using a vacuum system composing a SCROLLVAC 3S plus dry scroll vacuum pump and a TURBOVAC 90i turbomolecular pump (Leybold GmbH, Cologne, Germany). Once the internal pressure stabilized at 10^{-4} mbar, the valve was closed and the solution was thawed. These cycles were repeated until the absorbance at 404 nm reached to a steady-state level. Absorption spectra were acquired using a Cary 60 UV-VIS spectrophotometer (Agilent Technologies, Santa Clara, CA, USA).

Photoactivation of PAmCherry1 solution with a femtosecond laser

Photoactivation was performed using an amplified femtosecond double optical parametric amplifier laser system.[41,42] The pulse duration was 60 fs (FWHM) and the repetition rate was 1 kHz. The power of the 400 nm laser beam was set to 150 μW, which corresponds to 150 nJ per pulse. The laser beam was focused to a diameter of approximately 5 mm to fit within the quartz cuvette and directed onto the sample solution in a 1 cm pathlength quartz cuvette. The average laser fluence was calculated to be 0.76 $\mu J/cm^2$ per pulse. Photoactivation was monitored by acquiring absorption spectra using a Cary 60 UV-VIS spectrophotometer. Fluorescence emission spectra were recorded with a JASCO FP-8550 spectrofluorometer (Jasco Inc., Japan).

Supplementary tables

Table S1. Crystallographic analysis and refinement statistics for PAmCherry1

	Synchrotron	SFX
PDB entry	31AL	31JT
Data collection		
Space group	$P\ 2\ 2_1\ 2_1$	$P\ 2\ 2_1\ 2_1$
Unit cell		
a, b, c (Å)	39.36, 78.51, 145.4	39.36, 79.16, 145.4
α, β, γ (deg)	90.0, 90.0, 90.0	90.0, 90.0, 90.0
X-ray source	ESRF ID23-2	SACLA XFEL BL2(EH3)
Wavelength (Å)	0.8731	1.240
Resolution range (Å)	78.51– 1.50 (1.53– 1.50) [a]	24.80-1.63 (1.67-1.63)
Indexed lattices	N/A	62,078
Total reflections	580,172 (29,030)	60,243,001 (2,884,363)
Unique reflections	73,159 (3,567)	57,808 (3,823)
Multiplicity	7.9 (8.1)	1,042.1 (754.5)
Completeness (%)	99.9 (100.0)	100.0 (100.0)
I/σ(I)	13.1 (2.3)	8.1 (1.37)
Wilson B-factor (Å^2)	20.8	28.2
R_{meas} (%)	7.6 (90.5)	N/A
R_{split} (%)	N/A	8.4 (82.2)
$CC_{1/2}$	0.999 (0.865)	0.990 (0.493)
Refinement		
Resolution range *in refinement*	69.09-1.5 (1.52-1.50)	24.80-1.63 (1.66-1.63)
Number of reflections used in refinement	73,048	57,735
Number of reflections used for R_{free}	3,773 (156)	2,851 (131)
R_{work} (%)	17.9 (27.5)	16.3 (57.3)
R_{free} (%)	22.2 (31.9)	19.4 (55.2)
Number of non-hydrogen atoms	4,592	4,486
Macromolecules	3,911	4,018
Ligands	151	139
Solvent	530	329
Number of protein residues	447	448
RMSD bond length (Å)	0.006	0.007

RMSD bond angle (deg)	0.93	1.00
Ramachandran plot		
Outliers (%)	0.00	0.00
Allowed (%)	1.59	1.82
Favored (%)	98.41	98.18
Rotamer outliers (%)	0.96	1.84
Clash score	5.26	5.90
Average B-factor (Å²)	28.91	30.42
Macromolecules	27.01	29.41
Ligands	38.02	29.40
Solvent	40.31	43.21

[a] Statistics for the highest resolution shell are shown between parentheses. N/A; not applicable

Table S2. Equilibration protocol used to generate QM/MM optimized structures of PAmCherry1.

Run	Calculation type	Restraints / kcal mol^{-1} Å^{-2}	Constant	Minimization steps or Simulation time	PBC	Timestep / fs
1	minimization (CPU)	non-solvent 500	V	1000 steps	Yes	-
2	minimization (GPU)	CRO region 500	V	10 000 steps	Yes	-
3	heating (GPU) 0-300 K	CRO region 500	V	20 ps	Yes	2
4	equilibration (GPU)	CRO region 500	P	1 ns	Yes	2
5	cooling (GPU) 300-50 K	CRO region 500	V	20 ps	Yes	2
6	minimization (GPU)	CRO region 500	V	10 000 steps	Yes	-
7	QM/MM minimization (CPU)	border 1000	V	max 1000 steps	No	-

Table S3. Composition of the QM region for each PAmCherry1 form during QM/MM structure optimization. Residue numbering follows the original PDB files.

Form	Residues included in the QM region
Hydroperoxy	chromophore, Phe65 (backbone C, O, CA, HA), Ser69 (backbone N,H, CA, HA); side chain: Glu215, Arg95, Arg197
Enolate	chromophore, Phe60 (backbone C, O, CA, HA), Ser69 (backbone N,H, CA, HA); side chain: Glu215, Arg95, Arg197

Table S4. Composition of the QM region for each PAmCherry1 form for QM/MM excited-state calculations. Residue numbering follows the original PDB files.

Form	Residues included in the QM region
Hydroperoxy – small	chromophore, Phe65 (backbone C, O, CA, HA), Ser69 (backbone N,H, CA, HA)
Hydroperoxy – big	chromophore, Phe65 (backbone C, O, CA, HA), Ser69 (backbone N,H, CA, HA), one water molecule; side chain: Glu215, Arg95, Arg197, Gln42, Asn70
Enolate – small	chromophore, Phe65 (backbone C, O, CA, HA), Ser69 (backbone N,H, CA, HA)
Enolate – big	chromophore, Phe65 (backbone C, O, CA, HA), Ser69 (backbone N,H, CA, HA), three water molecules; side chain: Glu215, Arg95, Arg197, Gln42

Table S5. Vertical excitation energies and oscillator strengths of the QM/MM-optimized hydroperoxy form of PAmCherry1, calculated for the small QM region (Table S4) at the CC2/def2-TZVP level.

State	$\Delta E(S_0 \rightarrow S_n)$ / eV	f
1	3.788	0.000
2	4.044	0.007
3	4.606	0.031
4	4.691	0.036
5	4.877	0.019
6	4.974	0.035
7	5.136	0.020
8	5.179	0.011
9	5.323	0.010
10	5.440	0.001
11	5.644	0.003
12	5.742	0.004
13	5.757	0.004
14	5.796	0.005
15	5.911	0.010

Table S6. Vertical excitation energies and oscillator strengths of the QM/MM-optimized enolate form of PAmCherry1, calculated for the small QM region (Table S4) at the CC2/def2-TZVP level.

State	$\Delta E(S_0 \rightarrow S_n)$ / eV	f
1	3.285	0.861
2	4.005	0.004
3	4.594	0.012
4	4.657	0.001
5	4.810	0.035
6	4.927	0.012
7	5.018	0.011
8	5.129	0.009
9	5.265	0.030
10	5.291	0.033
11	5.619	0.002
12	5.651	0.004
13	5.789	0.012
14	5.821	0.004
15	5.833	0.019

Table S7. Vertical excitation energies and oscillator strengths of the QM/MM-optimized hydroperoxy form of PAmCherry1, calculated for the big QM region (Table S4) at the CC2/def2-TZVP level with the RVS approximation.

State	$\Delta E(S_0 \rightarrow S_n)$ / eV	f
1	3.732	0.001
2	4.061	0.006
3	4.530	0.023
4	4.631	0.042
5	4.794	0.021
6	4.816	0.012
7	4.863	0.009
8	4.968	0.038
9	5.080	0.011
10	5.230	0.013

Table S8. Vertical excitation energies and oscillator strengths of the QM/MM-optimized enolate form of PAmCherry1, calculated for the big QM region (Table S4) at the CC2/def2-TZVP level with the RVS approximation.

State	$\Delta E(S_0 \rightarrow S_n)$ / eV	f
1	3.335	0.815
2	3.728	0.018
3	4.343	0.000
4	4.579	0.012
5	4.655	0.006
6	4.724	0.020
7	4.839	0.009
8	4.941	0.044
9	4.968	0.022
10	5.186	0.006

Supplementary figures

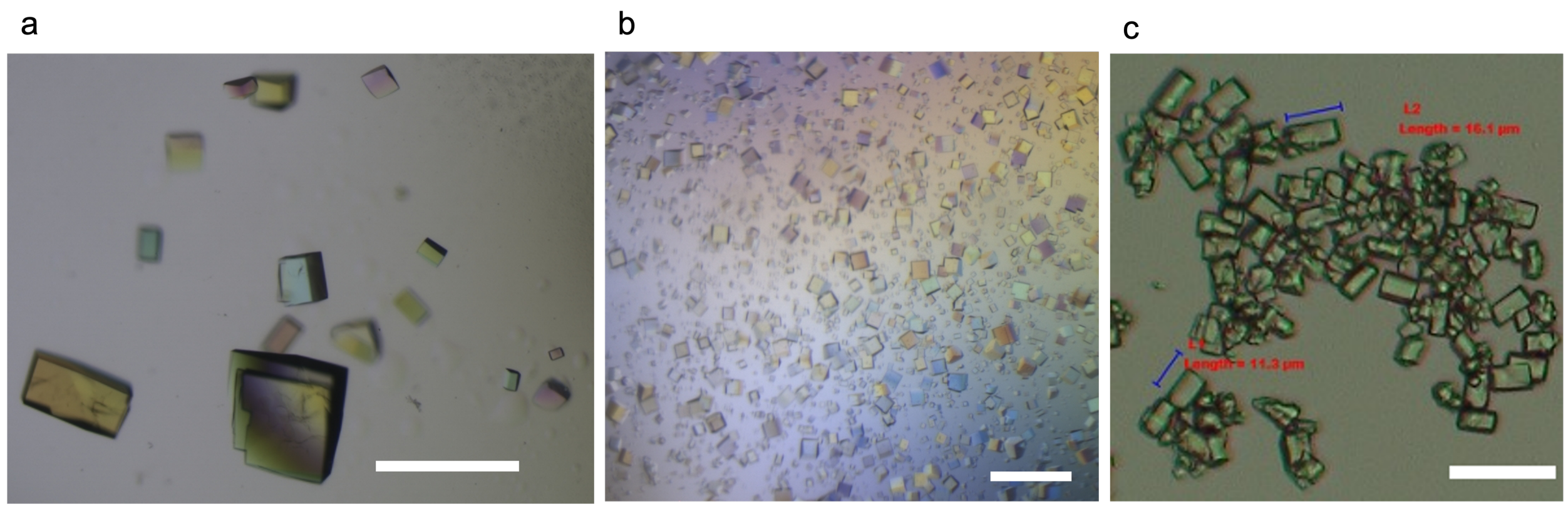

Figure S1. PAmCherry1 crystals used for X-ray crystallography. (a) Crystals grown by the hanging-drop vapor diffusion method (1 µL scale) used for synchrotron diffraction. (b) 10 µL batch crystallization, microseeded using crushed crystals from (a). (c) Large-scale batch crystallization (550 µL scale tubes) microseeded from (b) to prepare microcrystal slurries for SFX and TR-SFX. All crystals are grown at 20 ºC. The scale bar indicates 200 µm (a,b) and 30 µm (c).

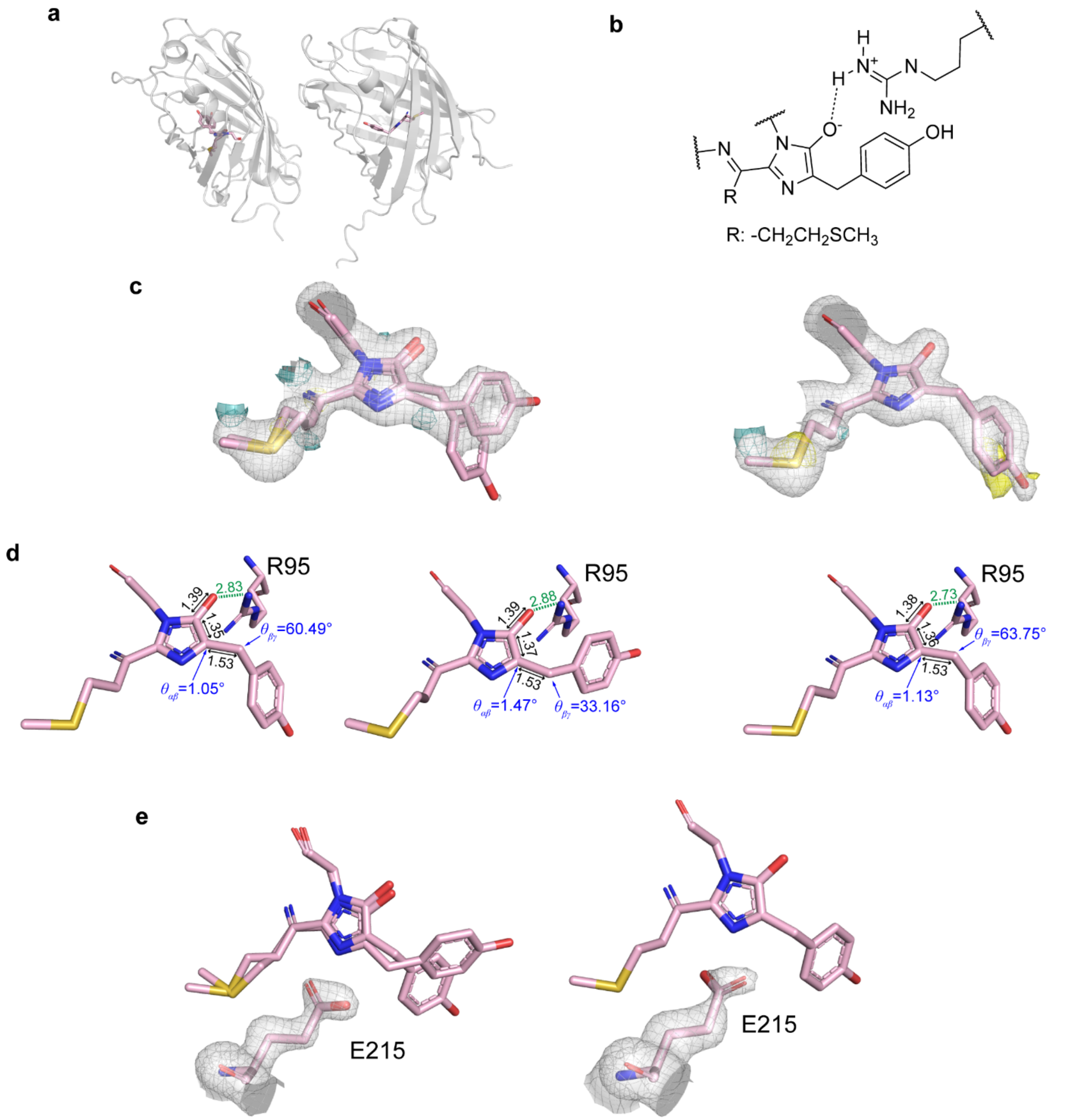

Figure S2. Synchrotron radiation structure of non-photoactivated PAmCherry1. (a) Overall structure of non-photoactivated PAmCherry1 is shown in cartoon representation. Chromophores are depicted with light pink sticks; oxygen, nitrogen, and sulfur atoms colored red, blue, and yellow, respectively. (b) Chemical structure of the enolate form chromophore, including the hydrogen bond with the R95 side chain. (c) 2mF_{obs}-DF_{cal} (gray) and mF_{obs}-DF_{cal} (yellow, negative; teal, positive) electron density maps around the chromophore refined with the enolate model for monomer A (left; two conformers) and monomer B (right), contoured at 1.0 and ±3.0σ, respectively. (d) Geometric analyses of the chromophore environment: the deviation of the Cα67-Cβ67 bond orientation relative to plane of the imidazolinone ring ($\theta_{\alpha\beta}$), the out-of-plane bending of Cβ67−Cγ67 ($\theta_{\beta\gamma}$), and bond lengths of Cα67-C67, C67-O67, and Cα67−Cβ67. The side chain of R95 and the distance between a terminal nitrogen of the guanidinium moiety and O67 are indicated. (left) minor conformer monomer A, (center) major conformer monomer A, (right) monomer B (e) 2mF_{obs}-DF_{cal} map (1.0σ) around E215 located near the chromophore. (left) monomer A, (right) monomer B.

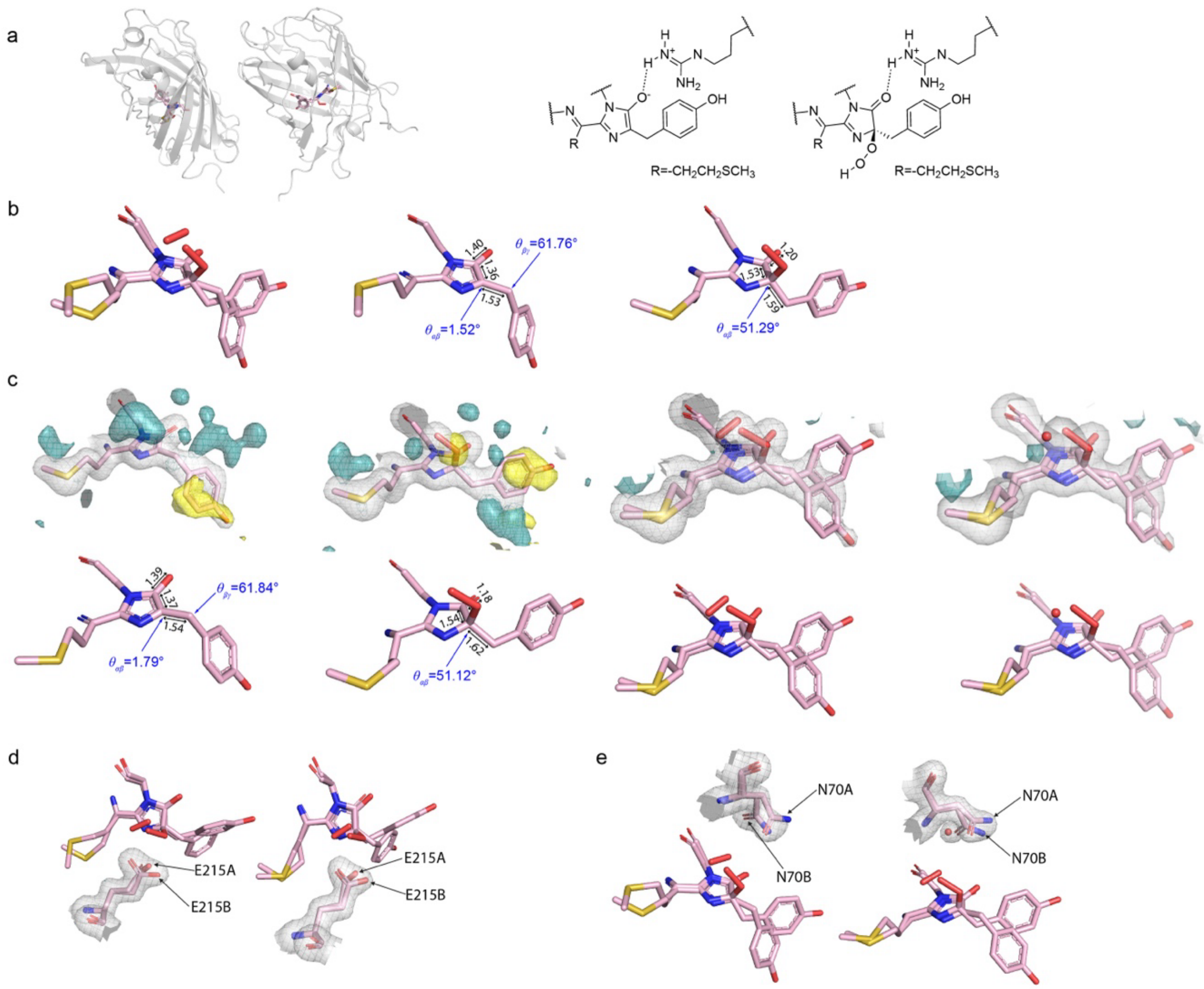

Figure S3. Crystal structure of non-photoactivated PAmCherry1 determined by SFX. (a) Overall structures of monomers A and B are shown in cartoon representation. Chromophores are depicted as light pink sticks. The chemical structures of the enolate and hydroperoxy form chromophores are shown on the right. (b) Best-fit model of monomer A obtained by refinement with a two-state model comprising hydroperoxy and enolate form chromophores, with refined occupancies of 73% and 27%, respectively. Geometric analyses of the chromophore environment are shown: $\theta_{\alpha\beta}$, bond orientation relative to the plane of the imidazolinone ring; $\theta_{\beta\gamma}$, out-of-plane bending of Cβ67−Cγ67. (c) Chromophore structures in monomer B refined using, from left to right, the enolate model, the hydroperoxy model, a two-state model with molecular oxygen, and a two-state model with a water molecule. In both two-state models, the refined occupancies of the enolate and hydroperoxy forms were 51% and 49%, respectively. The 2mF_{obs}-DF_{cal} (gray) and mF_{obs}-DF_{cal} maps (teal, positive; yellow, negative) are contoured at 1.0σ and ±3.0σ, respectively. Geometric analyses of the chromophore environment in the dual model refinement are provided below the maps: $\theta_{\alpha\beta}$, bond orientation relative to the plane of the imidazolinone ring; $\theta_{\beta\gamma}$, out-of-plane bending of Cβ67−Cγ67. From left to right: enolate form chromophore, hydroperoxy form chromophore, overlap of both chromophore structures in the two-state model with molecular oxygen or the two-state model with a water molecule. (d) E215 side chain near the chromophore with 2mF_{obs}-DF_{cal} map (1.0σ) for monomer A (left) and monomer B (right). Both conformers of the side chain are indicated. (e) N70 side chain near the chromophore with 2mF_{obs}-DF_{cal} map (1.0σ) for monomer A (left) and monomer B (right). Both conformers of the side chain are indicated.

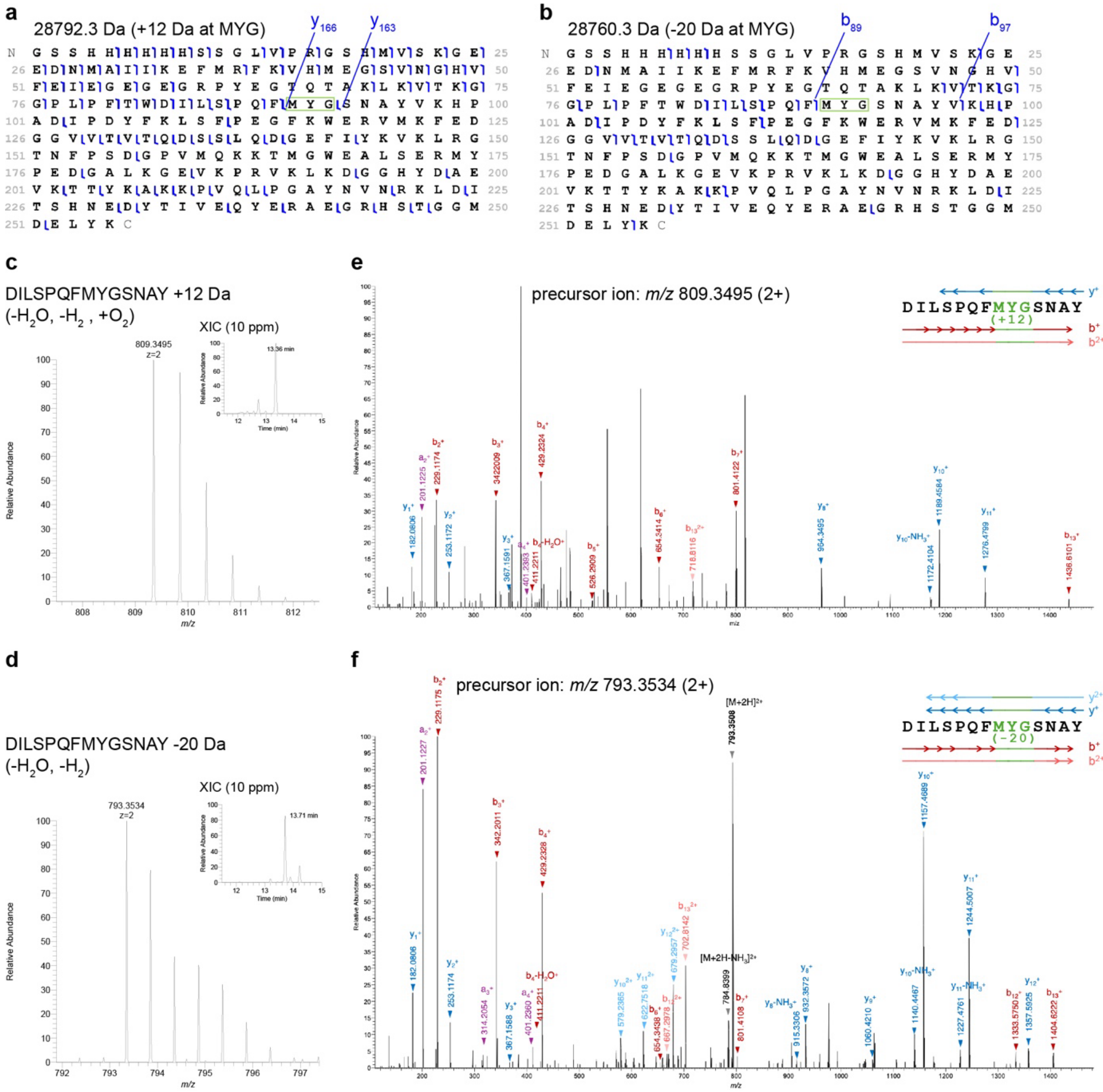

Figure S4. MS analysis of the non-photoactivated PAmCherry1. (a, b) Top-down MS/MS analysis. Fragmentation maps of the two predominant peaks with intact masses of 28792.3 Da (a) and 28760.3 Da (b) (Figure 2). Precursor ions at 15+ charge state were selected and fragmented via HCD. Green frames highlight the chromophore-forming amino acid residues. Detected backbone cleavage sites are indicated by symbols representing b-ions and y-ions. All observed fragment ions are consistent with a +12 Da modification (a) and a -20 Da modification (b) at the chromophore. The +12 Da modification was localized to the MYG motif based on the y_{166} and y_{163} ions, while the -20 Da modification was localized to the MYGSNAYV region based on the b_{89} and b_{97} ions. (c,d) MS1 spectra of two distinct modified species of chromophore-containing chymotryptic peptides. The peptides were observed at retention times of 13.36 min (c) and 13.71 min (d). The isotopic clusters correspond to the +12 Da species and the -20 Da species, respectively. Insets show XIC of respective ions (±10 ppm). (e,f) MS/MS analyses of the chromophore-containing chymotryptic fragments. Precursor ions at *m/z* 809.3495 (2+) (e) and *m/z* 793.3534 (2+) (f) were selected. The observed sequence ions are labeled as b- (red) and y-ions (blue). Arrows on the peptide sequence (top right in each panel) indicate the observed b- and y-ions. Detection of a shifted y_8 ion together with unmodified b_7 and y_3 ions localized the modification to the MYGS motif in both species. The fragment ions are consistent with the +12 Da (e) and -20 Da (f) modifications localized at the chromophore.

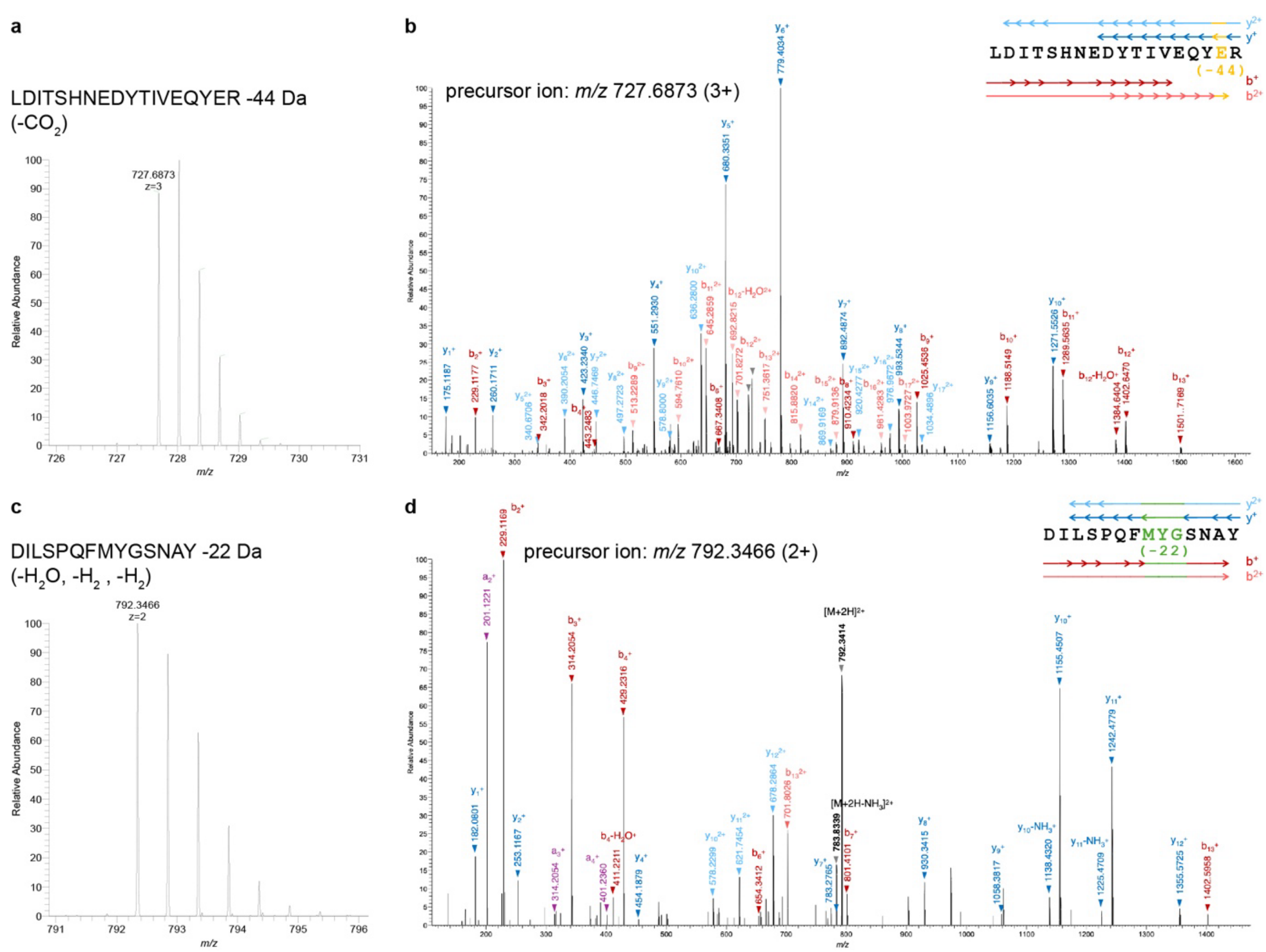

Figure S5. LC-MS analysis of the photoactivated PAmCherry1. (a) Detection of Kolbe decarboxylation at E215 upon the photoactivation. The MS1 spectrum of the tryptic peptide containing E215 exhibits a -44 Da shift relative to the native sequence, corresponding to the loss of carbon dioxide. (b) MS/MS analysis of precursor ion at *m/z* 727.6873 (3+). E215 is highlighted in yellow on the peptide sequence. The detection of shifted y_2 and b_{17} ions, together with unmodified y_1 and b_{16} ions locates the decarboxylation site to E215. (c) Detection of $C\alpha67{=}C\beta67$ double bond formation in the chromophore. The MS1 spectrum of the chymotryptic peptide containing the mature chromophore exhibited a -22 Da shift relative to the theoretical mass, that corresponds to a 34 Da decrease from the hydroperoxy intermediate. It is consistent with the release of hydrogen peroxide (H_2O_2, 34 Da) due to the oxidative dehydrogenation of the chromophore. (d) MS/MS analysis of the chymotryptic peptide of the photoactivated chromophore. The precursor ion at *m/z* 792.3466 (2+) was selected. Detection of a shifted y_7 ion with unmodified b_7 and y_4 ions localizes the modification to the MYG motif.

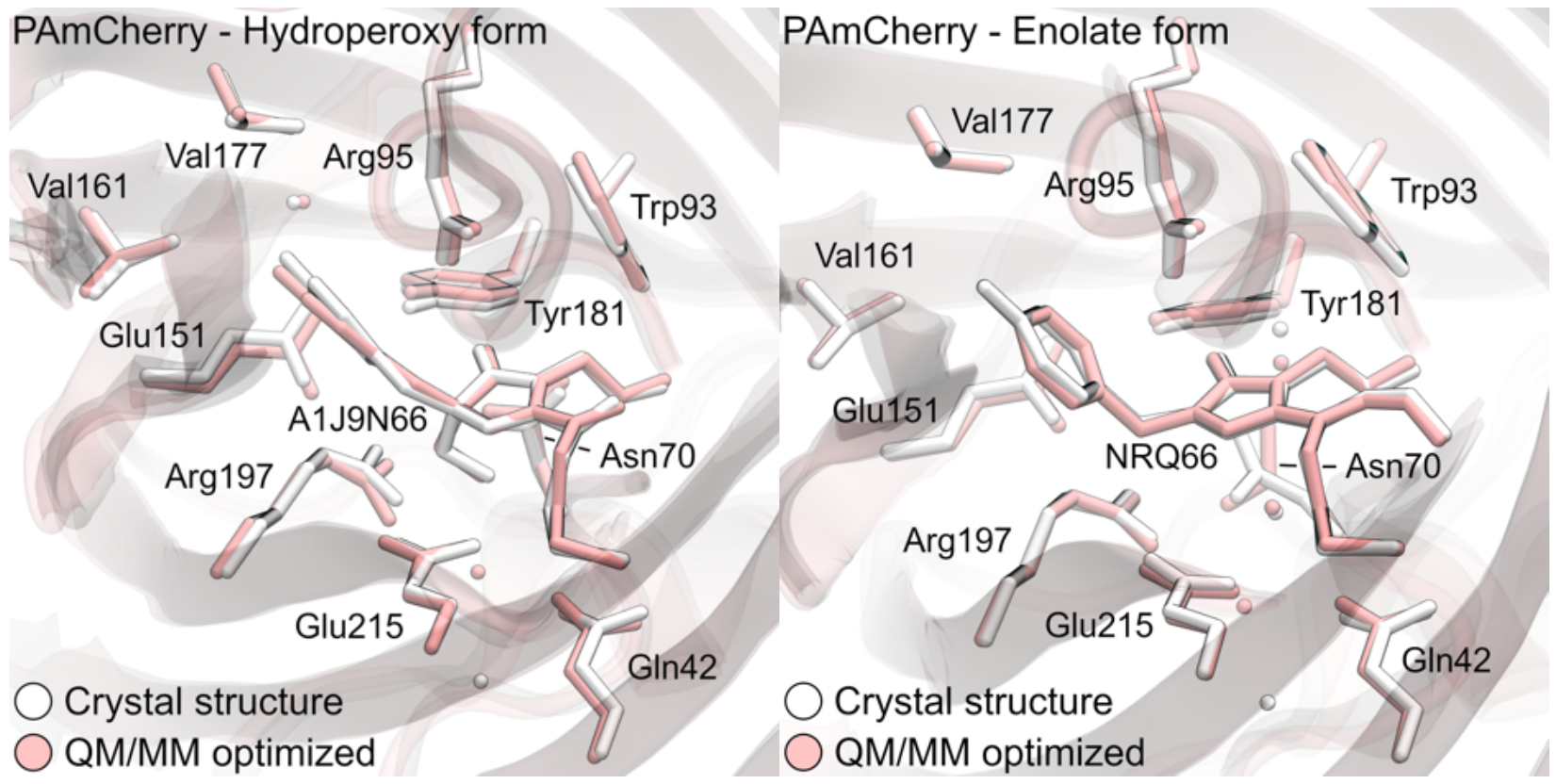

Figure S6. Comparison of QM/MM optimized (pink) and crystal (white) structures of PAmCherry1 in the hydroperoxy (left) and enolate (right) forms. The chromophore and nearby amino acid residues are shown as sticks and nearby water molecules as spheres. Hydrogen atoms are omitted for clarity. After QM/MM geometry optimization of PAmCherry1 in both the hydroperoxy and enolate forms, the overall structure of the chromophore pocket remained similar to the starting crystal structures. The only notable change in both cases was a displacement of a water molecule by approximately 3 Å, allowing it to form hydrogen bonds with Arg197 and Glu215. This observation suggests that this water molecule may occupy multiple positions or that not all structural waters were resolved in the crystallographic models. A similar water displacement was observed previously in our study of the fluorescent proteins LSSmKate1, PSLSSmKate, and LSSmOrange.[28]

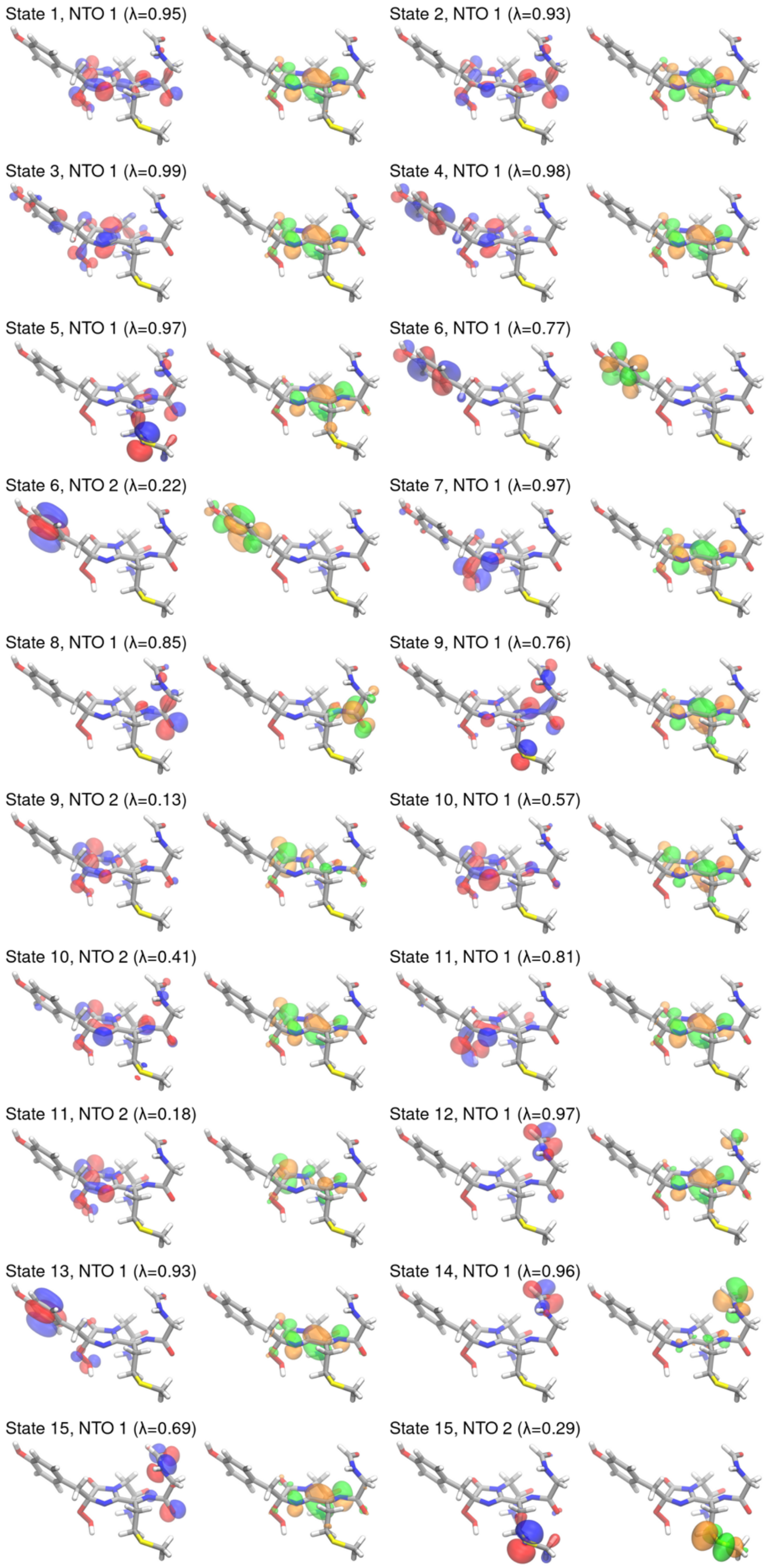

Figure S7. Most significant hole (red, blue) and electron (orange, green) natural transition orbitals (NTOs) of PAmCherry1 hydroperoxy form excited states, calculated with CC2/def2-TZVP, corresponding to Table S5.

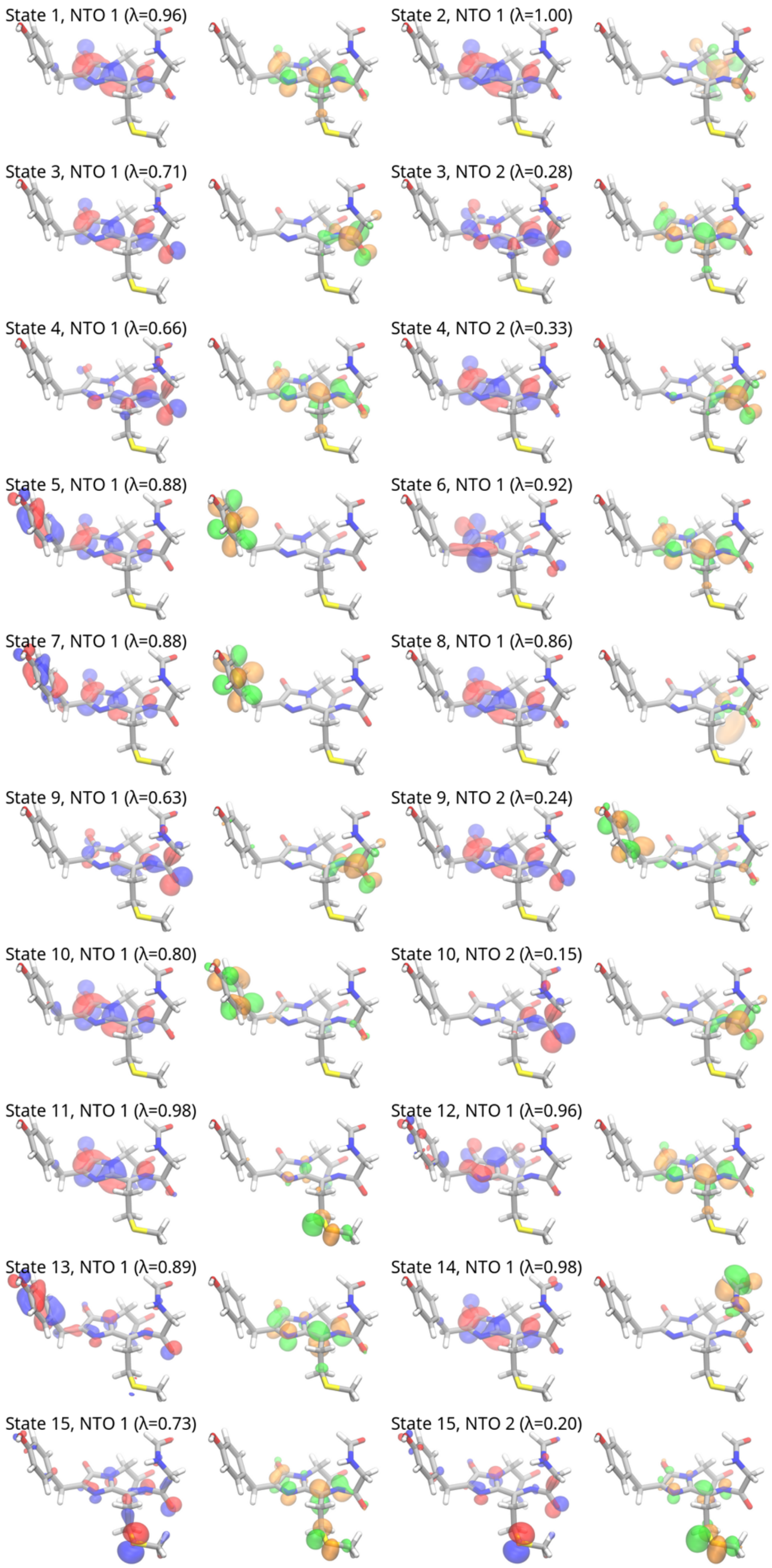

Figure S8. Most significant hole (red, blue) and electron (orange, green) natural transition orbitals (NTOs) of PAmCherry1 enolate form excited states, calculated with CC2/def2-TZVP, corresponding to Table S6.

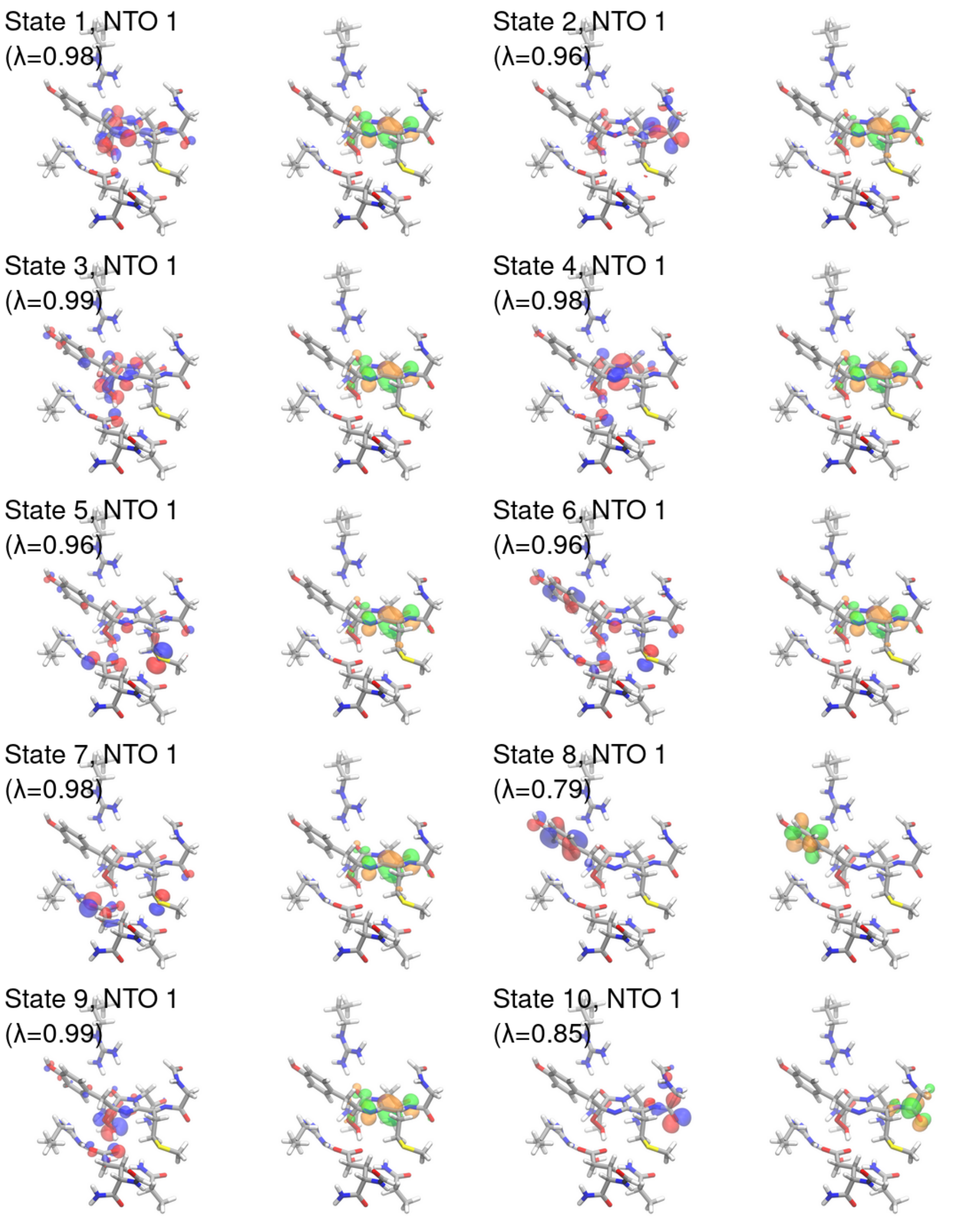

Figure S9. Most significant hole (red, blue) and electron (orange, green) natural transition orbitals (NTOs) of PAmCherry1 hydroperoxy form excited states, calculated with RVS-CC2/def2-TZVP and a big QM region, corresponding to Table S7.

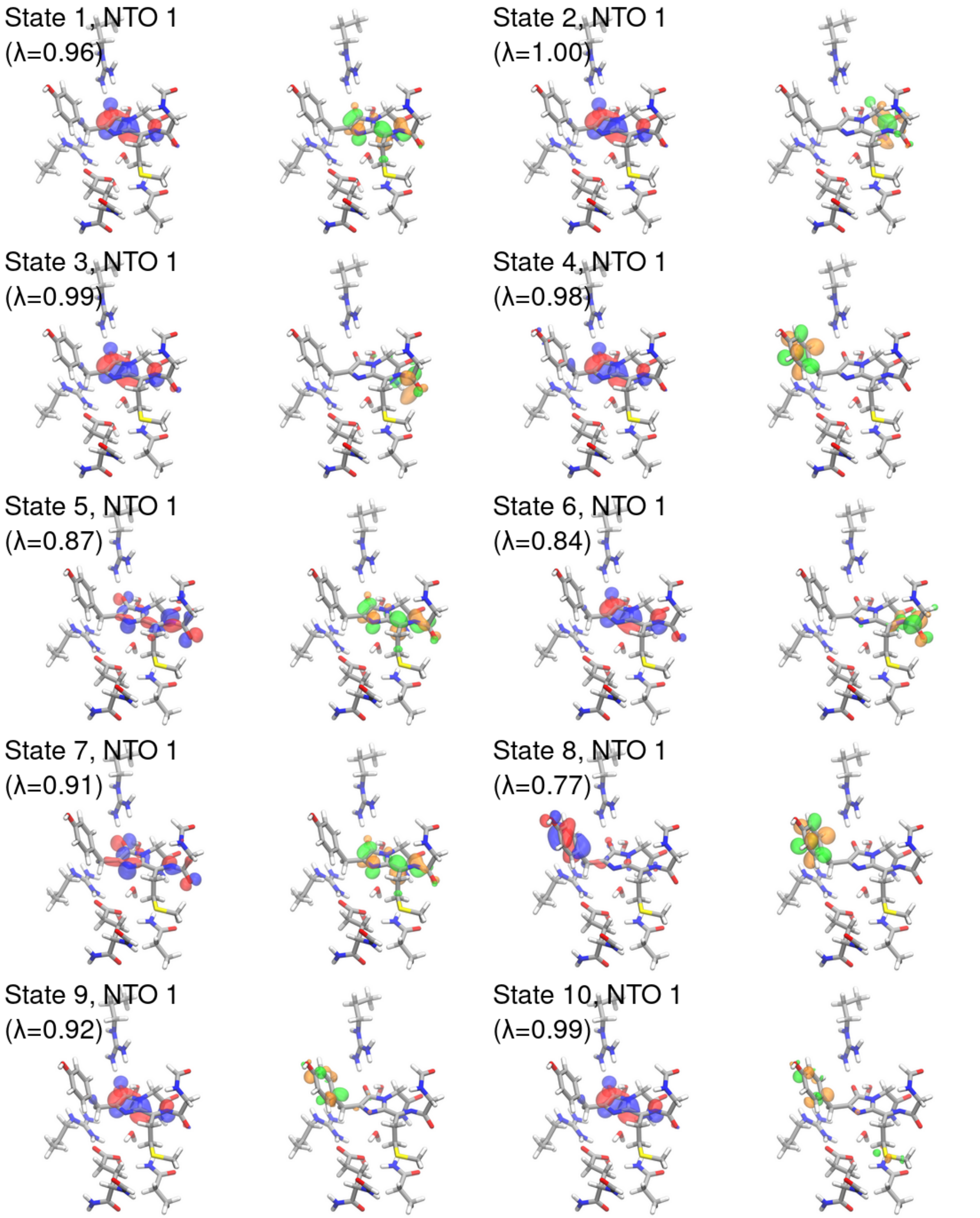

Figure S10. Most significant hole (red, blue) and electron (orange, green) natural transition orbitals (NTOs) of PAmCherry1 enolate form excited states, calculated with RVS-CC2/def2-TZVP and a big QM region, corresponding to Table S8.

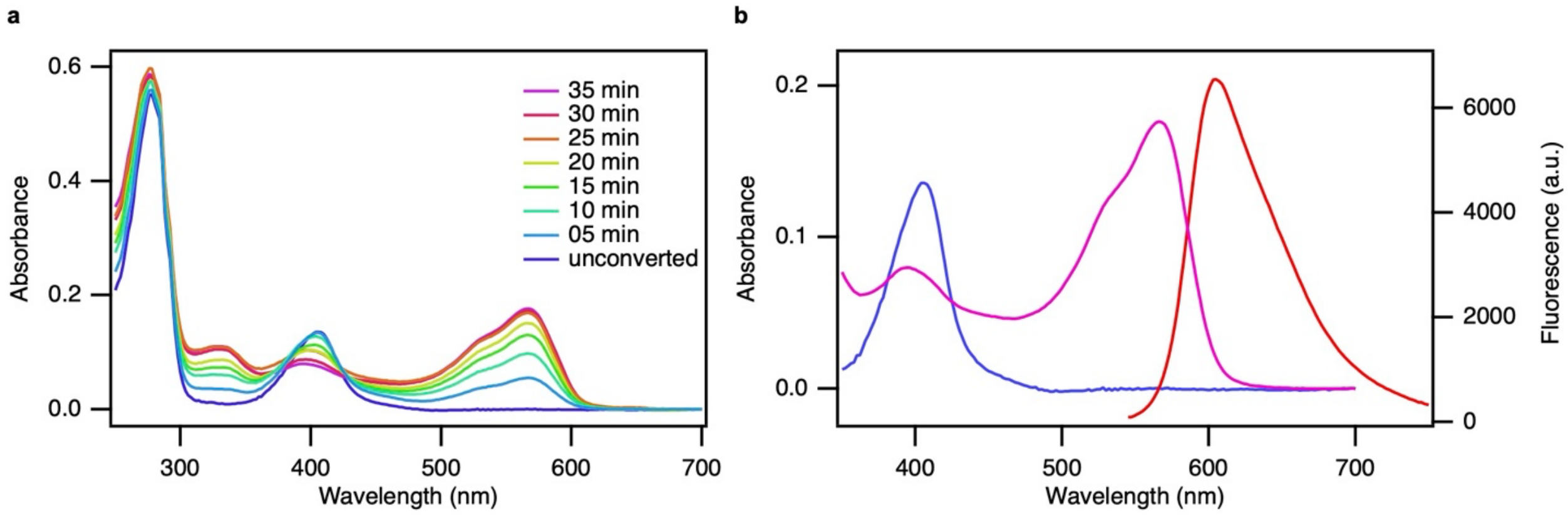

Figure S11. PAmCherry1 photoactivation by illumination with a fs-pulse laser. (a) Protein solution in a 1-cm cuvette was illuminated with a 400-nm fs-pulsed laser at 150 µW (150 nJ per pulse), and the absorption spectra were recorded over time. The absorption band peaking at 564 nm is characteristic for photoactivated PAmCherry1. (b) Absorption spectrum of photoactivated PAmCherry1 (magenta) and emission spectrum excited at 540 nm (red). The sample illuminated for 35 min with the fs-pulse laser was used for spectral measurements. The absorption spectrum of the non-photoactivated sample (blue) is overlaid.

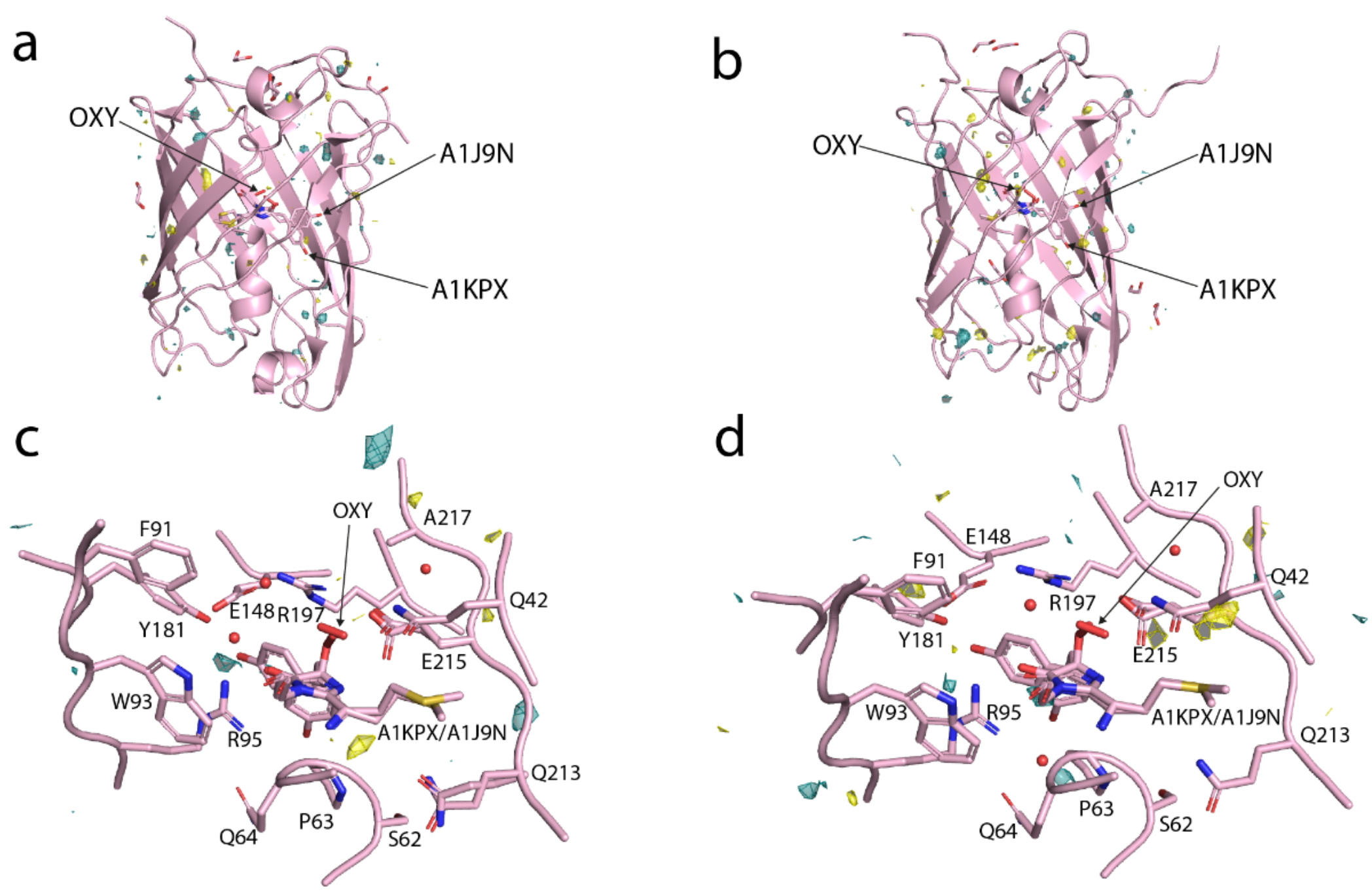

Figure S12. No differences were observed between the SFX dataset of non-photoactivated PAmCherry1 (complete dark) and the interleaved dark dataset of the TR-SFX experiment. (a, b) Fourier difference maps over monomer A (a) and monomer B (b), showing the chromophores and the oxygen molecules. (c, d) Fourier difference maps zoomed in on the chromophore pockets in monomer A (c) and monomer B (d). All maps are a $F_{obs}^{interleaved\ dark}$-$F_{obs}^{complete\ dark}$ Fourier difference maps (q-weighted) at 2.50 Å contoured at ±3 r.m.s.d. (yellow: negative electron density; teal: positive electron density; light pink: dark state structure).

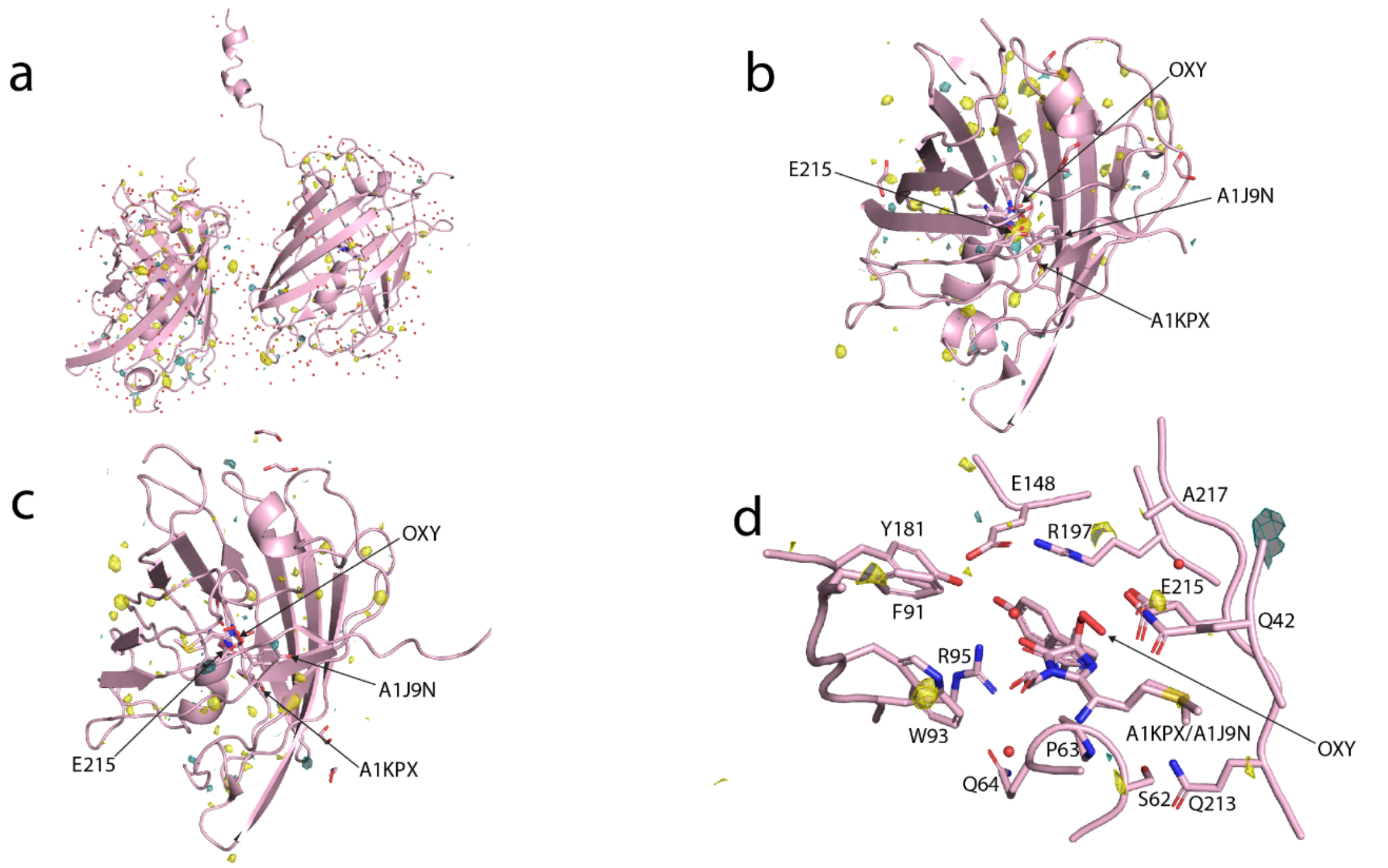

Figure S13. Pump-probe TR-SFX illustrates the PAmCherry1 photoinduced chromophore maturation in monomer A. (a) Fourier difference map over the two monomers in the asymmetric unit (left: monomer A; right monomer B). (b, c) Fourier difference maps over monomer A (b) and monomer B (c) with the chromophores, the oxygen molecules and E215. (d) Fourier difference map zoomed in on the chromophore pocket of monomer B. No abstraction of the hydroperoxide moiety and no Kolbe decarboxylation of E215 were observed in this monomer. All maps are a $F_{obs}^{light\text{-}800ps}$-$F_{obs}^{dark}$ Fourier difference maps (q-weighted) at 2.50 Å contoured at ±3 r.m.s.d. (yellow: negative electron density; teal: positive electron density; light pink: dark state structure).

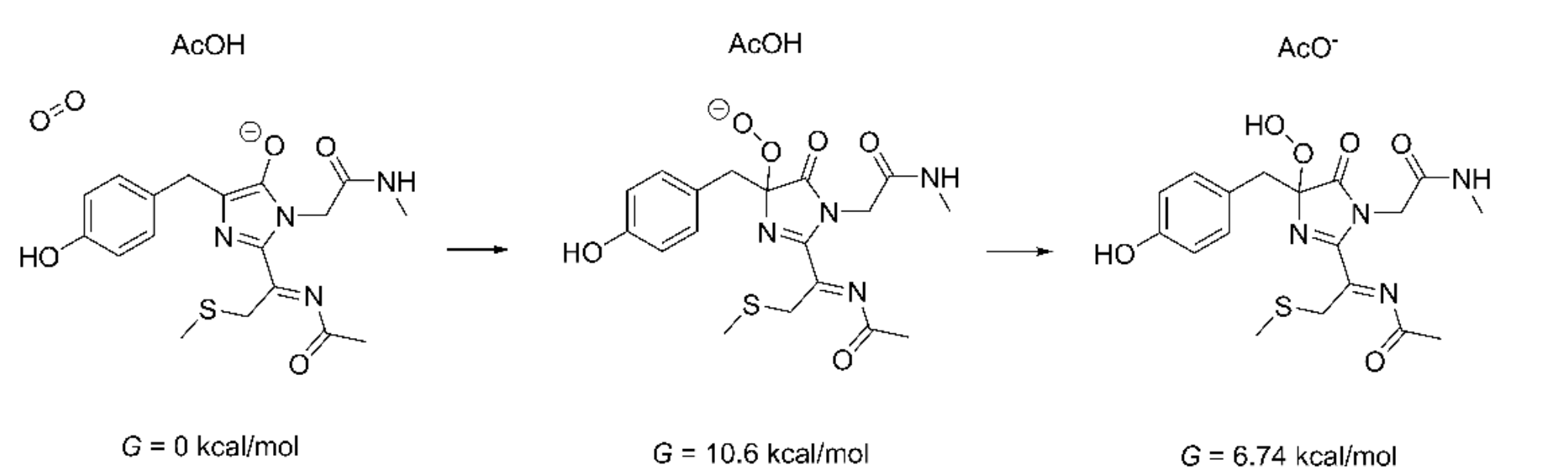

Figure S14. Thermodynamic cycle used to estimate the relative free energies of the hydroperoxy and enolate forms of the PAmCherry chromophore. Values were computed at the B3LYP/def2-TZVP/CPCM(chloroform) level at 298.15 K.

References

1. Seol, H. S. *et al.* Decarboxylation via a Higher Electronic Excited State Drives LSSmOrange Photoconversion. *ACS Phys. Chem. Au* **6**, 534–551 (2026).
2. Subach, F. V. *et al.* Photoactivatable mCherry for high-resolution two-color fluorescence microscopy. *Nat. Methods* **6**, 153–159 (2009).
3. Nanao, M. *et al.* ID23-2: an automated and high-performance microfocus beamline for macromolecular crystallography at the ESRF. *J. Synchrotron Radiat.* **29**, 581–590 (2022).
4. Evans, P. R. & Murshudov, G. N. How good are my data and what is the resolution? *Acta Crystallogr. Sect. D Biol. Crystallogr.* **69**, 1204–1214 (2013).
5. Zeldin, O. B., Gerstel, M. & Garman, E. F. RADDOSE-3D : time- and space-resolved modelling of dose in macromolecular crystallography. *J. Appl. Crystallogr.* **46**, 1225–1230 (2013).
6. Tono, K. *et al.* Beamline, experimental stations and photon beam diagnostics for the hard x-ray free electron laser of SACLA. *New J. Phys.* **15**, 083035 (2013).
7. Tono, K. *et al.* Diverse application platform for hard X-ray diffraction in SACLA (DAPHNIS): application to serial protein crystallography using an X-ray free-electron laser. *J. Synchrotron Radiat.* **22**, 532–537 (2015).
8. Kameshima, T. *et al.* Development of an X-ray pixel detector with multi-port charge-coupled device for X-ray free-electron laser experiments. *Rev. Sci. Instrum.* **85**, (2014).
9. Sugahara, M. *et al.* Hydroxyethyl cellulose matrix applied to serial crystallography. *Sci. Rep.* **7**, 703 (2017).
10. Shimazu, Y. *et al.* High-viscosity sample-injection device for serial femtosecond crystallography at atmospheric pressure. *J. Appl. Crystallogr.* **52**, 1280–1288 (2019).
11. Nakane, T. *et al.* Data processing pipeline for serial femtosecond crystallography at SACLA. *J. Appl. Crystallogr.* **49**, 1035–1041 (2016).
12. White, T. A. *et al.* CrystFEL : a software suite for snapshot serial crystallography. *J. Appl. Crystallogr.* **45**, 335–341 (2012).
13. Gevorkov, Y. *et al.* XGANDALF – extended gradient descent algorithm for lattice finding. *Acta Crystallogr. Sect. A Found. Adv.* **75**, 694–704 (2019).
14. Powell, H. R. The Rossmann Fourier autoindexing algorithm in MOSFLM. *Acta Crystallogr. Sect. D Biol. Crystallogr.* **55**, 1690–1695 (1999).
15. Duisenberg, A. J. M. Indexing in single-crystal diffractometry with an obstinate list of reflections. *J. Appl. Crystallogr.* **25**, 92–96 (1992).
16. Evans, P. R. & Murshudov, G. N. How good are my data and what is the resolution? *Acta Crystallogr. Sect. D Biol. Crystallogr.* **69**, 1204–1214 (2013).
17. Emsley, P. & Cowtan, K. Coot : model-building tools for molecular graphics. *Acta Crystallogr. Sect. D Biol. Crystallogr.* **60**, 2126–2132 (2004).
18. Adams, P. D. *et al.* PHENIX : a comprehensive Python-based system for macromolecular structure solution. *Acta Crystallogr. Sect. D Biol. Crystallogr.* **66**, 213–221 (2010).
19. Moriarty, N. W., Grosse-Kunstleve, R. W. & Adams, P. D. electronic Ligand Builder and Optimization Workbench (eLBOW): a tool for ligand coordinate and restraint generation. *Acta Crystallogr. Sect. D Biol. Crystallogr.* **65**, 1074–1080 (2009).
20. De Zitter, E., Coquelle, N., Oeser, P., Barends, T. R. M. & Colletier, J.-P. Xtrapol8 enables automatic elucidation of low-occupancy intermediate-states in crystallographic studies. *Commun. Biol.* **5**, 640 (2022).
21. Liebschner, D. *et al.* Polder maps: improving OMIT maps by excluding bulk solvent. *Acta Crystallogr. Sect. D Struct. Biol.* **73**, 148–157 (2017).
22. Winn, M. D. *et al.* Overview of the CCP 4 suite and current developments. *Acta Crystallogr. Sect. D Biol. Crystallogr.* **67**, 235–242 (2011).
23. Subach, F. V. *et al.* Photoactivation mechanism of PAmCherry based on crystal structures of the protein in the dark and fluorescent states. *Proc. Natl. Acad. Sci.* **106**, 21097–21102 (2009).
24. Subach, F. V. *et al.* Photoactivation mechanism of PAmCherry based on crystal structures of the protein in the dark and fluorescent states. *Proc. Natl. Acad. Sci. U. S. A.* **106**, 21097–21102 (2009).
25. Case, D. A. *et al.* AmberTools. *J. Chem. Inf. Model.* **63**, 6183–6191 (2023).
26. Tian, C. *et al.* ff19SB: Amino-Acid-Specific Protein Backbone Parameters Trained against Quantum Mechanics Energy Surfaces in Solution. *J. Chem. Theory Comput.* **16**, 528–552 (2020).
27. Jorgensen, W. L., Chandrasekhar, J., Madura, J. D., Impey, R. W. & Klein, M. L. Comparison of simple potential functions for simulating liquid water. *J. Chem. Phys.* **79**, 926–935 (1983).
28. Čivić, J., Mizuno, H. & Harvey, J. N. Photoinduced decarboxylation in fluorescent proteins: charge-transfer states and structure–function relationship. *Phys. Chem. Chem. Phys.* **27**, 24777–24788 (2025).
29. Grimme, S., Brandenburg, J. G., Bannwarth, C. & Hansen, A. Consistent structures and interactions by density functional theory with small atomic orbital basis sets. *J. Chem. Phys.* **143**, (2015).
30. Case, D. A.; Aktulga, H. M.; Belfon, K.; Ben-Shalom, I. Y.; Berryman, J. T.; Brozell, S. R.; Cerutti, D. S.; III, T. E. C.; Cisneros, G. A.; Cruzeiro, V. W. D.; Darden, T. A.; Duke, R. E.; Giambasu, G.; Gilson, M. K.; Gohlke, H.; Goetz, A. W.; Harris, R.;, P. A. Amber 2022. at (2022).

31. Götz, A. W. *et al.* Routine Microsecond Molecular Dynamics Simulations with AMBER on GPUs. 1. Generalized Born. *J. Chem. Theory Comput.* **8**, 1542–1555 (2012).
32. Salomon-Ferrer, R., Götz, A. W., Poole, D., Le Grand, S. & Walker, R. C. Routine Microsecond Molecular Dynamics Simulations with AMBER on GPUs. 2. Explicit Solvent Particle Mesh Ewald. *J. Chem. Theory Comput.* **9**, 3878–3888 (2013).
33. Le Grand, S., Götz, A. W. & Walker, R. C. SPFP: Speed without compromise—A mixed precision model for GPU accelerated molecular dynamics simulations. *Comput. Phys. Commun.* **184**, 374–380 (2013).
34. Götz, A. W., Clark, M. A. & Walker, R. C. An extensible interface for QM/MM molecular dynamics simulations with AMBER. *J. Comput. Chem.* **35**, 95–108 (2014).
35. Neese, F., Wennmohs, F., Becker, U. & Riplinger, C. The ORCA quantum chemistry program package. *J. Chem. Phys.* **152**, (2020).
36. Olsen, J. M. H. PyFraME: Python Tools for Fragment-Based Multiscale Embedding. at https://doi.org/https://doi.org/10.5281/zenodo.1168860 (2018).
37. TURBOMOLE V7.8 2023, a Development of University of Karlsruhe and Forschungszentrum Karlsruhe GmbH, 1989-2007, TURBOMOLE GmbH, since 2007. at https://www.turbomole.org.
38. Balasubramani, S. G. *et al.* TURBOMOLE: Modular program suite for ab initio quantum-chemical and condensed-matter simulations. *J. Chem. Phys.* **152**, (2020).
39. Marefat Khah, A., Karbalaei Khani, S. & Hättig, C. Analytic Excited State Gradients for the QM/MM Polarizable Embedded Second-Order Algebraic Diagrammatic Construction for the Polarization Propagator PE-ADC(2). *J. Chem. Theory Comput.* **14**, 4640–4650 (2018).
40. Schwabe, T. *et al.* PERI–CC2: A Polarizable Embedded RI-CC2 Method. *J. Chem. Theory Comput.* **8**, 3274–3283 (2012).
41. Kennes, K. *et al.* Photophysical Investigation of Cyano-Substituted Terrylenediimide Derivatives. *J. Phys. Chem. B* **118**, 14662–14674 (2014).
42. Fron, E. *et al.* Revealing the Excited-State Dynamics of the Fluorescent Protein Dendra2. *J. Phys. Chem. B* **117**, 2300–2313 (2013).
